# Effective treatment of petroleum oil–contaminated wastewater using activated sludge modified with magnetite/silicon nanocomposite

**DOI:** 10.1007/s11356-023-26557-6

**Published:** 2023-05-01

**Authors:** Nidal M. Zabermawi, Ebtesam El Bestawy

**Affiliations:** 1 Department of Biological Sciences, Faculty of Science, King Abdulaziz University, P.O. Box 42805, Jeddah, 21551 Kingdom of Saudi Arabia; 2 Department of Environmental Studies, Institute of Graduate Studies and Research, Alexandria University, 163 Horria Ave. El-Shatby, P.O. Box 832, Alexandria, Egypt

**Keywords:** Activated sludge, Effective treatment, Magnetite/silicon nanocomposite, Modification, Petroleum oil, Refinery wastewater

## Abstract

The study aimed to optimize the treatment of oil refinery–contaminated wastewater through modification of the well-established activated sludge process with new nanocomposite (NC) materials to produce high-quality treated effluents for potential reuse. Refinery wastewater samples were collected from one of the major oil refineries, Alexandria, Egypt, where the operation, performance, and efficiency of the current activated sludge (AS) unit were evaluated for 6 consecutive months. Two AS bench scale PVC basins were constructed. Magnetite nanoparticles (Fe_3_O_4_ NPs) and magnetite silica (Fe_3_O_4_/silica) nanocomposite (NC) were prepared and characterized. Bioremediation trials were carried out in a sequential batch mode using Fe_3_O_4_/silica NC–modified AS and control (unmodified AS). The proposed treatment produced high-quality effluents in a very short time (2 h) despite the very high initial pollutant concentration accompanied with a reduction in the produced sludge. The highest removal of TSS, TDS, BOD, COD, and OG from raw industrial wastewater recorded 78.33, 3.6, 87.65, 85.17, and 92.92% compared to 55.3, 12.6, 50.0, 40.22, and 56.84%, respectively, achieved by the unmodified AS unit. The results confirmed that integration of the AS treatment with nanomaterial composite is highly effective, promising, and economic for the treatment of highly toxic and complicated industrial wastewater such as petroleum refinery effluents.

**Supplementary Information:**

The online version contains supplementary material available at 10.1007/s11356-023-26557-6.

## Introduction

Water is one of the most plentiful natural resources that covers 70% of the earth’s surface and undoubtedly is the most valuable resource on earth that is directly linked to the survival of living organisms including humans (Omer et al. 2022). However, only about 1% of that resource is available for human consumption, most of which has been exhausted and is likely to be continuously contaminated by a variety of organic and inorganic pollutants because of rapid industrialization and the increasing world population (Anjum et al. 2019; Saleem and Zaidi 2020). The oil refining industry is one of the most important industries economically, strategically, and environmentally. It converts crude oils into over 2500 refined products (gasoline, kerosene, jet fuel, etc.) and is remarkably affecting water resources worldwide through using large quantities of water (246–341 L/barrel of crude oil) for cooling processes, desalting processes, and stripping steam. Consequently, it is generating large volumes of wastewater (0.4- to 1.6-fold the processed crude oil volume) that must be properly treated (Kuyukina et al. 2020). Petroleum refinery effluents (PREs) differed from refinery to refinery due to variations in plant configuration and type of crude oil processed. They contain a variety of biodegradable, inhibitory, and toxic organic and inorganic pollutants mainly light aliphatic fractions and polycyclic aromatic petroleum hydrocarbon. They also contain nitrogen, sulfur compounds, phenols, and heavy metals; therefore, they must be regulated to meet a specific quality standard before discharging into the open environment (Elmobarak et al. 2021; Malollari et al. 2022).

The variety and complexity of wastewaters generated by the oil industry are enormous and encompass a wide range of priority pollutants at varied concentrations that negatively impact the ecosystems since they are generally harmful and more persistent in the environment. They release unwanted toxins into the air, water sources, and soil all over the world. Refinery wastewater endangers the received aquatic resources (drinking water, groundwater resources, open oceans, or rivers) and endangers human health since it contains a mixture of complex contaminants that are reported to be toxic, mutagenic, and carcinogenic in nature. In addition to air pollution, PREs can affect crop production due to soil pollution, destructing the natural landscape and causing safety problems due to the oil burner (Salem and Thiemann 2022). Therefore, PREs result from the manufacturing process, cleaning activities, or from leakages representing the main challenge to treatment and safe discharge. Wastewater treatment systems have been designed to remove biological oxygen demand (BOD), chemical oxygen demand (COD), suspended solids (SS), pathogenic organisms, nitrogen, and phosphorus, but the removal of these substances alone is no longer sufficient to protect the quality of the environment. More sustainable practices, in particular, wastewater treatment at very efficient levels aiming at reuse is the main target of oil refineries (Simões et al. 2020).

In order to comply with the environmental regulations and safely discharged, the treatment of refinery wastewater, including highly toxic organic contaminants, must adopt integrated approaches since the common conventional chemical and biological treatment technologies are not sufficient to achieve the required effluent quality standards. Manipulation of the astonishing characteristics of the emerging new materials has led to remarkable enhancement in many applications, among which wastewater treatment technologies. Integration of such materials with traditional treatment technologies enables operators to produce high-quality, recyclable treated effluents at very high efficiency and low cost, as well as conserve the very limited water resources and protect the environment’s biotic and abiotic elements from the expected adverse effects (Kuyukina et al. 2020). Activated sludge (AS) is a very common treatment technology in the oil refinery industry. Modification of AS with  multifunctional and highly efficient nanomaterials and composites provides affordable advanced solutions for the treatment of PREs and requires no large infrastructures or centralized systems (Fiorenza et al. 2020; Ajith et al. 2021; Kamali et al. 2019). Due to their small sizes (< 100 nm in at least one dimension), nanomaterials acquire magnificent physicochemical, optical, and biological properties. Because of their higher surface area-to-volume ratio, high reactivity, and strong sorption, they are widely used for remediation purposes, sensing and detection, and pollution prevention applications (El-Monaem et al. 2021). The ability to behave as colloids, high absorption, and interaction capabilities enable nanomaterials to upgrade wastewater treatment to levels not achievable by conventional technologies (Gautam et al. 2013; Muzammil et al. 2019). Moreover, integration with nanomaterials leads to reducing energy consumption and saving costs in treatment systems (Sallam et al. 2018; Abd El-Monaem et al. 2022).

Application of nanoparticle materials in the manufacturing of polymeric and inorganic membranes greatly enhances their characteristics such as small pore size properties, permeability control, fouling resistance, and other advanced properties depending on the selected polymers (Wei 2018; Ahmad et al. 2022), which subsequently improves the filtration systems to remove toxic metals, organic, and inorganic solutes as well as disinfecting water/wastewater from disease-causing microbes (Barhate and Ramakrishna 2007). The main aim of the present study was to optimize the treatment efficiency of an oil refinery contaminated wastewater through modification of the well-established AS process with new nanocomposite materials to produce high-quality treated effluents for potential reuse.

## Materials and methods

### Wastewater sampling

Wastewater samples were collected from an oil refinery that handles different types of crude oil blends based on conventional treatment using the activated sludge (AS) process. Industrial wastewater samples were collected from the equalization basin after API gravity separators and before entering the DAF unit in the treatment path inside the oil refinery. API gravity separators remove free oil and solid particles while in the equalization basins, the effluents are mixed with spent caustic and sour water striper which minimizes fluctuations in wastewater temperature and contaminant concentrations. Samples were collected at different time schedules to examine wastewater with different pollution strengths as a result of different production capacities and schemes. Raw sanitary wastewater samples were used during the startup stage of the proposed AS laboratory units were collected from the inlet of the domestic wastewater treatment plant (DWWTP). Wastewater samples were subjected to physical and chemical characterization before and after treatment to define their pollution strength and calculate the treatment efficiency.

### Evaluation of the efficiency of the oil refinery AS system

The levels of the major contaminants in the raw and AS-treated wastewater at the selected oil refinery were obtained from the daily routine analysis in the refinery labs. These data were used to calculate the removal efficiency of four major contaminants (TSS, BOD, COD, and oil content) in order to evaluate the operation, performance, and efficiency of the current AS system at the refinery.

### Development of the bench scale–activated sludge unit (sequential batch reactor, SBR)

#### Construction

Two bench scale units were constructed using polyvinyl chloride (PVC) basins at 25 × 25 × 25 cm dimensions each. The unit was supplied with 3 taps; one is working as an inlet for the raw wastewater at one side, and two are working as outlets for the treated water at two elevated vertical levels (Supplementary material S1). The basin is divided into 2 rooms with a barrier at 5 cm from the wall side of the inlet tap and 5 cm away from the bottom (S2). This barrier was set to slow down and adjust the wastewater flow into the center of the basin as well as reduce water turbulence.

#### Startup of the activated sludge culture

The bench scale units were started using 300-g wet AS each, collected from the aerated basin of the AS unit at one of the largest oil refineries. This sludge was supplied by 4 L of oily wastewater collected from the equalization basin of the same refinery to which 2 L of raw sanitary wastewater was added at the starting point to help the culture growth and reduce the toxic effect of industrial wastewater. Each unit was continuously aerated to help the growth and maturation of the AS culture, especially at the starting stage. After 24 and 72 h, each unit was supplied with 2 L of raw sanitary wastewater to enrich the cultures under continuous aeration (S3).

#### Monitoring of activated sludge maturation

The AS culture was monitored daily for 43 days during preparation of the proposed lab. AS unit to define its maturation and start the treatment process. Monitoring parameters included DO, pH, TSS, sludge volume (SV_30_), mixed liquor suspended solids (MLSS), and sludge volume index (SVI). After 12 days from the starting point of the AS culture, aeration was stopped for 30 min, and 3 L of clear water was decanted and replaced by 6 L of fresh raw sanitary wastewater. In order to refresh the AS culture and maintain bacterial growth, this procedure was repeated on the 18th and 26th day of AS culture preparation process where 2 and 1.5 L were replaced, respectively. In addition, 300-g industrial AS was added to each basin on the 17th day of the culture age. After the incubation period and maturation of the AS culture, the proposed system was used in the treatment of oil-contaminated wastewater with and without modification using nanocomposite.

### Synthesis and characterization of the nanocomposite material (NCM)

Magnetite/silicon nanocomposite was synthesized, characterized, and examined for the enhancement of AS treatment for the decontamination of oil refinery wastewater.

#### Synthesis of magnetite nanoparticles (Fe_3_O_4_ NPs)

Synthesis of Fe_3_O_4_ NPs was carried out using a hydrothermal or solvothermal reaction according to the following steps:

1. FeCl_3_·6H_2_O (27 g) and sodium acetate trihydrate (120 g) were completely dissolved in 500 mL of ethylene glycol under vigorous magnetic stirring for 30 min (Madrid et al. 2014).
  1 $${\mathrm{FeCl}}_3\cdot6{\mathrm H}_2{\mathrm O}_{\;_{(\mathrm s)}}+3\mathrm C{\mathrm H}_3{\mathrm{COONa}}_{\;(\mathrm s)}+{\mathrm C}_2{\mathrm H}_6{{\mathrm O}_2}_{\;(\mathrm L)}\rightarrow\mathrm{Fe}{{(\mathrm{OH})}_3}_{\;(\mathrm s)}+3{\mathrm{NaCl}}_{\;(\mathrm s)}+3\mathrm C{\mathrm H}_3\mathrm{COOH}\;_{(\mathrm L)}+{\mathrm C}_2{\mathrm H}_6{{\mathrm O}_{2\;}}_{(\mathrm L)}+3{\mathrm H}_2{\mathrm O}_{\;(\mathrm L)}$$
2. The obtained homogenous brown-yellow solution was transferred to a Teflon-lined stainless-steel autoclave and sealed to heat at 200 °C (close to the boiling point of ethylene glycol 197 °C) for 8 h after which the autoclave was left to cool to room temperature. In that reaction, ethylene glycol acts as a reducing agent to produce iron(II) hydroxide (Madrid et al. 2014).
  2 $$\mathrm{Fe}{(\mathrm{OH})}_{3(\mathrm s)}+{\mathrm C}_2{\mathrm H}_6{\mathrm O}_{2(\mathrm L)}\xrightarrow\bigtriangleup\mathrm{Fe}{(\mathrm{OH})}_{2(\mathrm s)}+{\mathrm C}_2{\mathrm H}_4{\mathrm O}_{(\mathrm L)}+{\mathrm H}_2{\mathrm O}_{(\mathrm L)}+{\mathrm{OH}^-}_{(\mathrm{aq})}$$
3. Iron(III) and iron(II) hydroxides from Eqs. 1 and 2, respectively, react in a 2:1 molar ratio at a higher temperature (190 °C) to form magnetite nanostructures. The resultant black magnetite particles were washed with ethanol 6 times to remove any impurities and improve drying characteristics, separated by centrifugation, and dried in vacuum at a 60 °C for 12 h (Zhang et al. 2019).
  3 $$2\;\mathrm{Fe}{(\mathrm{OH})}_{3\left(\mathrm s\right)}+\mathrm{Fe}{(\mathrm{OH})}_{2(\mathrm s)}\xrightarrow\bigtriangleup{\mathrm{Fe}}_3{\mathrm O}_{4(\mathrm s)}\downarrow+4{\mathrm H}_2{\mathrm O}_{(\mathrm l)}$$
4. The produced Fe_3_O_4_ NPs (7.2 g, 94% yield) were used in the synthesis of Fe_3_O_4_/silica nanocomposite material (NCM).

It should be noted that rapid stirring of the reaction mixture ensures a homogeneous temperature during the phase transformation of iron oxides, which is necessary to obtain a narrow size distribution of the Fe_3_O_4_NPs (Cha et al. 2013).

#### Synthesis of magnetic silica (Fe_3_O_4_/silica) nanocomposite

1. Treatment of 5.0-g Fe_3_O_4_ NPs (~ 300 nm in diameter) with 0.1-M HCl aqueous solution (250 mL) using ultrasonication for 10 min.
2. After sonication, Fe_3_O_4_ NPs were separated and washed with deionized water and then homogeneously dispersed in a mixture of ethanol (80 mL), water (20 mL), and concentrated ammonia aqueous solution (20 mL, 28 wt.%).
3. This was followed by the addition of tetraethyl orthosilicate (TEOS, 1.5 g, 0.144 mmol).
4. After stirring at room temperature for 6 h, the Fe_3_O_4_/silica microspheres were separated by an external magnet and washed with ethanol and water (Deng et al. 2008). Overall, a 5.6-g yield of Fe_3_O_4_@nSiO_2_ was obtained after air-drying.

#### Characterization of the nanocomposite materials (NCM)

Synthesized nanocomposite materials (NCM) were characterized using scanning (SEM) and transmission (TEM) electron microscope, Fourier transform infrared spectroscopy (FTIR), X-ray diffraction (XRD), energy-dispersive X-ray (EDX), and thermal gravimetric analysis (TGA) to explore their physical, chemical, and morphological properties. Morphology and elemental analysis of the prepared nanocomposite were investigated using SEM (JEOL JSM-IT 200, Japan) equipped with an energy-dispersive X-ray (EDX). TEM (JEOL, JEM 100 CX, Japan) was used to determine the particle size and morphology of the prepared nanocomposites. The crystal phase was examined using XRD (BRUKER D8 Advance Cu target, Germany), operating with CuKα radiation (*λ* = 1.54 A°) generated at 40 kV and 40 mA. The functional groups as well as molecular interactions between the adsorbed molecules and the NPs of the magnetic nanocomposite were examined using FTIR (Model 8400 S, Shimadzu, Japan). Thermal stability was determined using thermal gravimetric analysis (TGA-50 Shimadzu, Japan).

### Treatability studies using SBR

Three AS treatment trials were examined in the present study on oil-contaminated wastewater. Two trials were performed with the developed unmodified AS reactor, and another trial was performed using the developed AS reactor modified by Fe_3_O_4_/silica NCM. Treatability studies were performed to achieve the optimum conditions that lead to the highest removal efficiency of petroleum oil and other contaminants in the oily wastewater using AS system augmented with NCM. The 3 treatment trials using the proposed bench scale AS reactor included (1) treatment of raw domestic wastewater, (2) treatment of oil refinery wastewater after API gravity separators and equalization process, and (3) treatment of raw oil refinery wastewater using the proposed AS reactor augmented with Fe_3_O_4_/silica NC (0.1 g/2 L of wastewater). The sequential batch reactor (SBR) treatment mode was adopted during the present study where the same reactor was used as an aeration basin (with aeration) and secondary clarifier (without aeration).

After the maturation of the culture in the proposed unmodified AS reactor, the first treatment trial was performed with domestic wastewater (DWW). The reactor was filled with about 6 L of DWW and left overnight to allow acclimatization between the wastewater and the AS culture under aeration. After 24 h, 1 L of wastewater was drawn while aeration was on to characterize the AS culture. Then aeration was stopped for 30–60 min (settling and clarification), after which, the treated sample was drawn from the upper outlet tap and characterized. This procedure was repeated for 12 days, and samples were collected at 24-h intervals. The same procedure was carried out using API-treated oil wastewater, and treatment was performed for 13 days with daily wastewater sampling. Since AS technology is a process designated to treat both domestic and industrial wastewater, the 2 previous trials were performed to evaluate the bioremediation capabilities of the proposed lab-scale unmodified AS reactor towards real domestic and industrial wastewater before incorporating the nanomaterials. Treatment of domestic wastewater, at this stage, enriches the AS culture, which reduces or prevents the toxic effects and shock loads that might result if oil refinery wastewater was applied directly to the AS reactor. Thus, it helps in enhancing the treatment efficiency of the proposed system and prepares the culture for dealing with the nanocomposite to produce high-quality effluent, the main focus of the present study.

In the third trial, oil refinery wastewater was treated using AS augmented with Fe_3_O_4_/silica nanocomposite. After filling the reactor with API-treated oil wastewater, Fe_3_O_4_/silica microsphere composite was added (0.05 g/L of wastewater) and left for 24 h to be integrated with AS bacteria under continuous aeration. AS culture and treated wastewater were characterized after the first 24-h exposure. This procedure was repeated for 8 days for treated wastewater samples that were collected daily. After sampling of the treated effluents during the treatment trials, the selected parameters were analyzed to determine their residual levels at each exposure time, and their removal efficiency was calculated to determine the effectiveness of the remediation process.

### Characterization of the raw and treated effluents

Quality parameters (temperature, pH, DO, TSS, TDS, BOD, COD, and OG) of the domestic and industrial wastewaters were characterized before and after the proposed treatment using the standard techniques described in the “Standard Methods for the Examination of Water and Wastewater” (Rice et al. 2017). Residual levels at each exposure time and their removal efficiency were calculated to determine the effectiveness of the remediation process according to the following equation:

4 $$\mathrm{Removal}\;\mathrm{efficiency}\;(\mathrm R\mathrm E\%)={\mathrm C}_0-\mathrm{RC}/{\mathrm C}_0\times100$$

where C_0_ is the initial concentration before treatment (zero time) and RC is the residual concentration after treatment at each exposure time.

## Results and discussion

### Evaluation of the efficiency of the oil refinery AS system

The oil refining industry is one of the most important industries economically, strategically, and environmentally. It is seriously and remarkably affecting water resources worldwide. They use large quantities of water for cooling, desalting processes, and stripping steam which in turn generates large volumes of wastewater that must be treated properly (Woodard and Curran Inc. 2011). Discharge of contaminants from petroleum industries forms residual COD, which has detrimental environmental consequences and is affecting all living organisms due to high oxygen demand and toxicity of the individual components in the wastewater. Thus, strict regulations are in place in terms of minimum levels allowable for their disposal (Elmobarak et al. 2021; Simões et al. 2020).

**Fig. 1:**
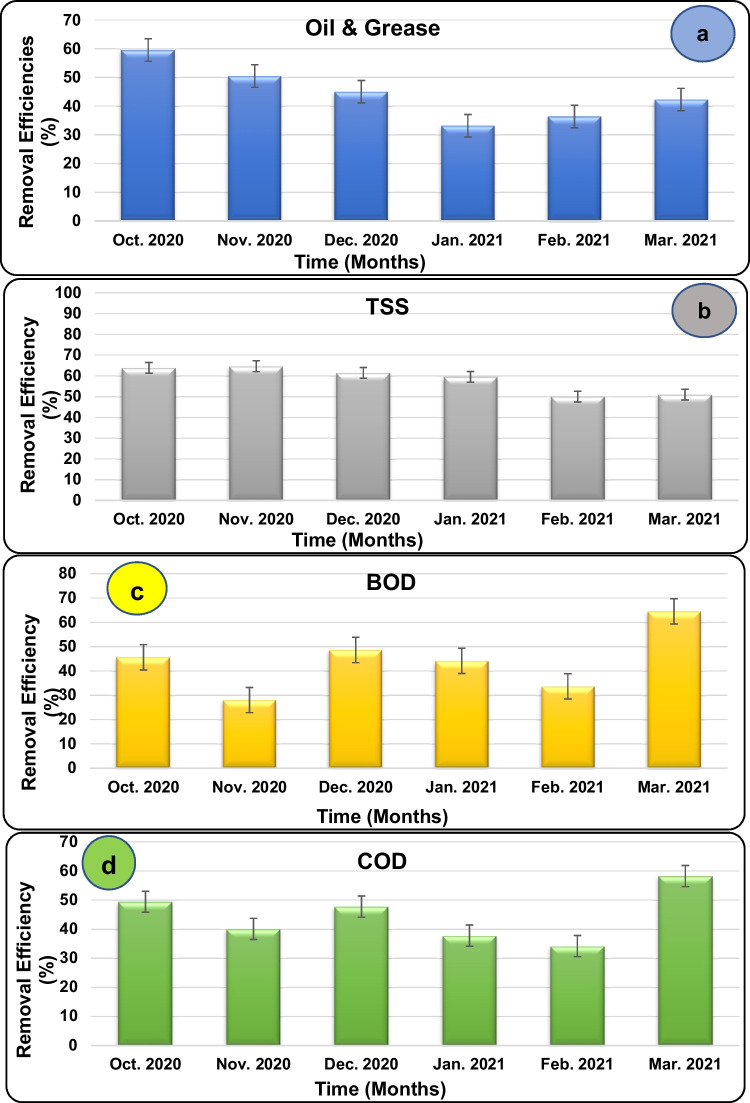
Removal Efficiencies (REs%) of the OG (**a**), TSS (**b**), BOD (**c**), and COD (**d**) in the effluent treated by the oil refinery AS system during the period October 2020–March 2021

Appendices 1–4 (S4–S7) represent residual concentration (RC, mg/L) and removal efficiency (RE%) of OG, COD, BOD, and TSS in the AS-treated effluents, respectively, at the selected oil refinery (Fig. 1a–d). As a general trend, the installed AS treatment unit at the oil refinery exhibited low to moderate RE% of the indicator quality parameters confirming its low efficiency and performance. Based on this fact, RCs of all the tested parameters in the final effluents were far exceeding their maximum permissible limits (MPLs) stated by the Egyptian environmental regulation law (No. 48/ 1982 Clause 52 Modified by Decree 92/2013) for safe discharge into non–freshwater open aquatic environments.

The average monthly RE of OG ranged between 33.2 ± 29.3 and 59.6 ± 7.7% (Fig. 1a) reaching the lowest RCs of 200.9 to 139.1 mg/L, which are 20.1 to 13.9-fold higher than its MPL (10 mg/L). Slightly higher TSS removal was recorded with an average monthly RE range of 50.0 ± 19.3– 64.6 ± 14.5% (Fig. 1b) reaching the lowest RCs of 100.8 to 99.9 mg/L, which are 1.99- to 2.0-fold higher than its MPL (50 mg/L). The average monthly RE of BOD ranged between 28.0 ± 8.1 and 64.5 ± 18.5% (Fig. 1c) reaching the lowest RCs of 557.8 to 427.1 mg/L, which are 9.3- to 7.1-fold higher than its MPL (60 mg/L). Finally, the average monthly RE of COD ranged between 34.1 ± 37.9 and 58.2 ± 20.1% (Fig. 1d) reaching the lowest RCs of 889.5 to 657.1 mg/L, which are 11.1- to 8.2-fold higher than its MPL (80 mg/L). The highest pollution load in the AS-treated effluent is the OG followed by BOD, COD, and finally TSS, respectively. Therefore, the evaluation of the on-site established AS plant confirmed low performance and effectiveness and indicated that it is not capable to produce effluents with quality to comply with the environmental regulations for safe discharge into open systems. Such evaluation is opposed to other workers who successfully showed high removal efficiencies for COD (94–95%), TOC (85–87%), and TSS (98–99%) by using activated sludge for decontamination of petroleum refinery wastewaters (Santo et al. 2013) and confirmed that the on-site established AS plant in the present study is not qualified for decontamination of the refinery effluent. It is also a highly recommended modification with nanocomposite materials to enhance treatment efficiency.

### Development of the bench scale–activated sludge unit (sequential batch reactor, SBR)

Bench scale AS unit was developed in order to investigate the effect of modification with Fe_3_O_4_/silica NCM to enhance the AS capability to handle the pollution load in the oil refinery influent and produce environmentally safe effluent. Table 1 represents the characterization of AS culture during the startup stage. DO and pH levels recorded ranges of 2.3–2.8 and 3.1–3.7 mg/L as well as 6.9–7.2 and 6.9–7.1 in the two aeration basins, respectively, at the starting stage of the biological treatment. DO levels showed a satisfactory amount of oxygen in the aeration basin. The values of pH started slightly acidic due to the formation of carbonic acid (H_2_CO_3_) that slightly increased with continuous operation recording a slight alkaline medium (pH ˃ 7) in the mature sludge stage.

**Table 1:** Activated sludge characterization during the startup and maturation stage

Time (days)	Unit (1)					Unit (2)									
	DO (mg/L)	pH	SV_30_ (ml/L)	MLSS (mg/L)	SVI (mg/mL)	DO (mg/l)		pH		SV_30_ (ml/L)		MLSS (mg/L)		SVI (mg/mL)	
3	2.5	6.95	94.0	1888**	49.788*	3.1*		6.88^*^		112		1750*		64.00	
4	3.1	6.90	96.0	1842	52.117	3.24		6.89		100		1773		56.402	
5	2.3*	6.89^*^	90.0^*^	1760	51.136	3.5		6.94		100		1786		55.991	
10	2.3	6.94	97.0	1750	55.429	3.74		6.92		95*		1799		52.807*	
11	2.4	6.99	100.0	1760	56.818	3.4		6.97		97		1792		54.129	
12	2.55	6.98	99.0	1775	55.775	3.3		6.99		99		1821		54.366	
13	Decantation of 3 L of clear water and addition of 6 L of fresh raw domestic wastewater														
	2.4	6.94	102.0	1820	56.044		3.6		6.94		103.53		1785		58.00
17	Addition of 300-g industrial activated sludge to each basin														
	2.8	6.92	105.0	1800	58.333		3.5		6.93		110		1824		60.307
18	Decantation of 2 L of clear water and addition of 2 L of fresh raw domestic wastewater														
	2.3	6.98	108.0	1811	59.636		3.1		6.99		104		1800		57.778
19	2.7	7.1	104.0	1824	57.018		3.2		7.01		100		1830		54.645
20	2.5	7.1	100.0	1830	54.645		3.25		7.00		110		1822		60.373
24	2.5	7.2	100.0	1833	54.555		3.16		7.12		114		1841**		61.923
26	Addition of 1.5 L of fresh raw domestic wastewater														
	2.7**	---	103.0	1840**	56.0		3.5**		7.1		107		1821		58.759
43	2.3	7.1**	125**	1623*	77.0**		3.5**		7.13		118**		1744		67.0**

^**^The highest value

^*^The lowest value

Also, during this stage, the average concentrations of MLSS recorded ranges of 1623–1888 and 1750–1841 mg/L in the two basins, respectively, which is considered suitable for operation conditions and must be fixed at this range to fix the sludge age. SVI that reflects sludge characters showed stable behavior with values ranging between 49.8 and 77.0 mL/mg in the first basin and between 52.8 and 67.0 mL/mg in the second basin. It is known that the optimum sludge volume index must range between 50 and 120 mL/mg; therefore, SVI of the developed bench scale AS reactors indicated good settleability and compatibility properties of the sludge. Based on these conditions, the startup stage ended after 43 days, and the developed AS units were considered mature and ready for treatability study.

### Characterization of Fe_3_O_4_/silica nanocomposite material (NCM)

#### Scanning and transmission electron microscopy (SEM and TEM)

**Fig. 2:**
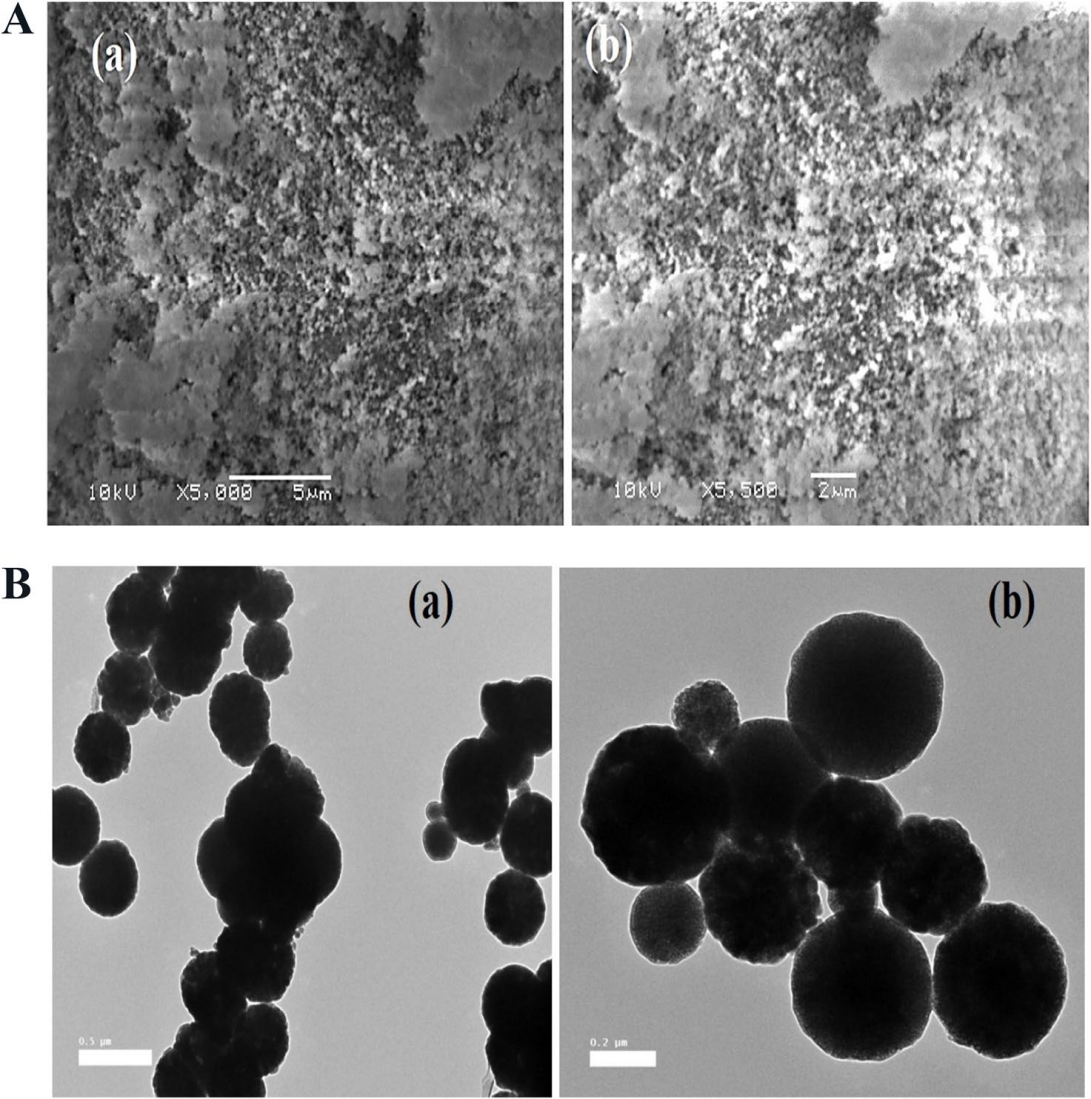
**a** SEM micrographs of Fe_3_O_4_/silica nanocomposite. **b** TEM micrographs of Fe_3_O_4_/silica nanocomposite

The morphology of Fe_3_O_4_/silica nanocomposites was investigated using the SEM technique (Fig. 2a). Fe_3_O_4_/silica are spherical shaped particles with some aggregations as a result of the magnetic properties of magnetite. The TEM micrograph (Fig. 2b) showed that Fe_3_O_4_/silica are spherical shaped particles with high size variation.

#### X-ray diffraction (XRD)

**Fig. 3:**
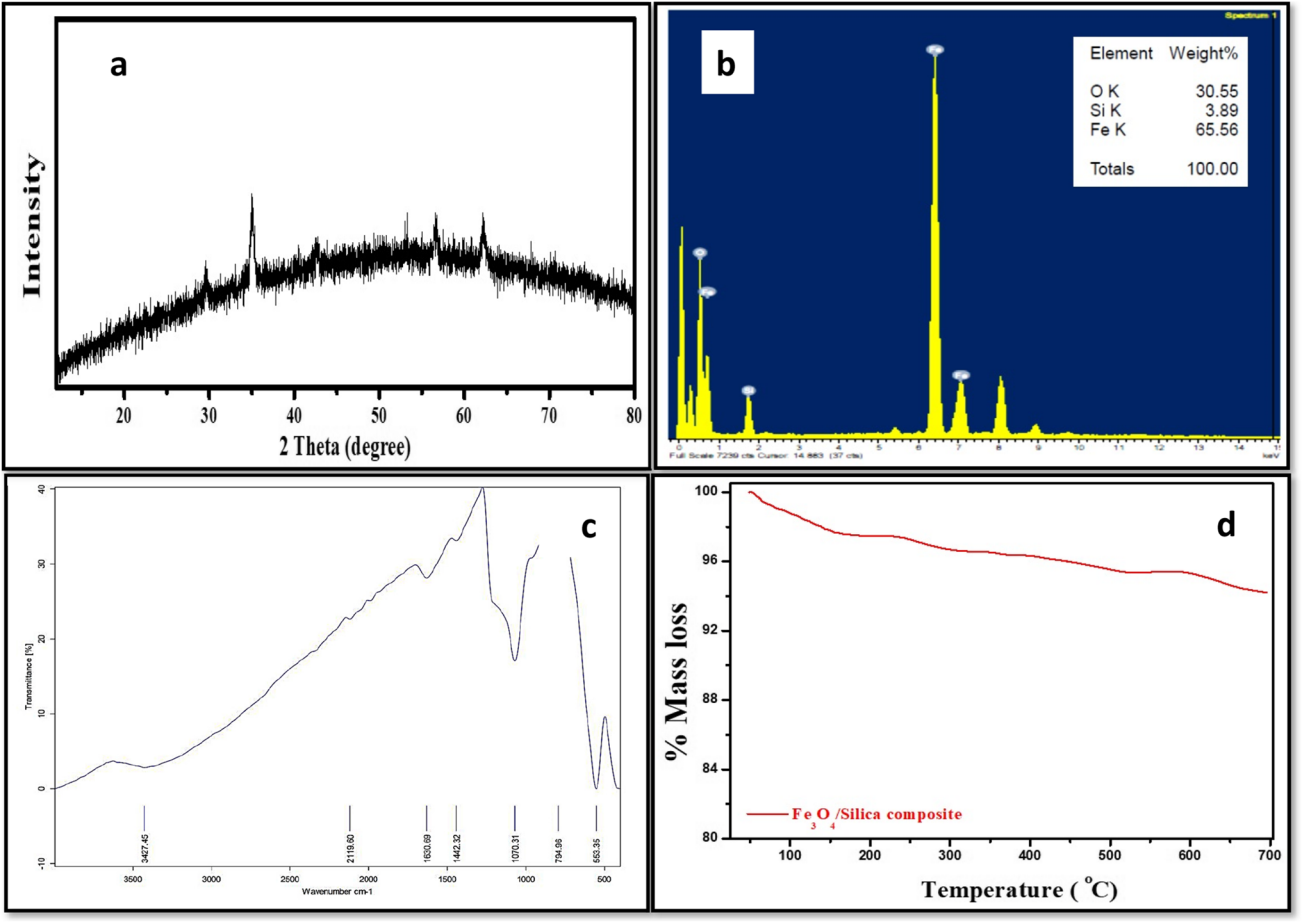
X-ray diffraction pattern (**a**), EDX micrograph (**b**), TGA curve (**c**), and FTIR spectrum (**d**) of Fe_3_O_4_/silica nanocomposite

The crystalline structure of Fe_3_O_4_/silica NC was investigated by the XRD diffraction pattern (Fig. 3a). The figure showed that the magnetite standard characteristic peaks at 2θ of 29.8°, 35.5°, 43°, 53.7°, 57.2°, and 62.7° are observed which are corresponding to the 220th, 311th, 400th, 511th, 422nd, and 440th crystalline planes, respectively. However, there are no characteristic peaks for silica reflecting its amorphous phase as well as the good coating of magnetite nanoparticles with silica. The diffraction peaks of magnetite are attributed to the cubic phase structure of magnetite according to JCPDS card, No. 01–089-0691. The XRD patterns of magnetite nanoparticles indicated that they are well crystallized.

#### Energy-dispersive X-ray spectroscopy (EDX)

The EDX analysis of Fe_3_O_4_/silica nanocomposite (Fig. 3b) showed that Si, O, and Fe are the main constituents of Fe_3_O_4_/silica with a mass percent of 30.6, 3.9, and 65.6, respectively, confirming the presence of both Fe_3_O_4_ and silica in the nanocomposite.

#### Fourier transform infrared spectroscopy (FTIR)

The FTIR analysis of both Fe_3_O_4_/silica nanocomposites (Fig. 3c) showed a band at 553 cm^−1^, which is the typical IR vibration/absorbance band induced by the structure Fe–O bond vibration of Fe_3_O_4_. The band at 1070 cm^−1^ is corresponding to Si–O–Si antisymmetric stretching vibrations, being indicative of the existence of SiO_2_ in the nanocomposite. Si–OH bond is confirmed by an IR absorption band at 794.96 cm^−1^. The broad absorption band at 3427 cm^−1^ and the band around 1630 cm^−1^ are due to OH bending and stretching vibrations of OH groups on the surface of iron oxide, which is attributed to the adsorbed water molecules.

#### Thermal gravimetric analysis (TGA)

The TGA diagram for Fe_3_O_4_/silica nanocomposite (Fig. 3d) showed that the first mass loss occurred at 50–200°C, caused by the loss of the water molecules absorbed in the nanocomposite surface. Then there was a small mass loss up to 700°C, reflecting the high stability of Fe_3_O_4_/silica nanocomposite where the total mass loss was found to be 6% only.

### Treatability studies using SBR

Three AS treatment trials were examined and included two with the developed unmodified AS reactor and one using AS reactor modified (augmented) by Fe_3_O_4_/silica nanocomposite (NC) to achieve optimum operation conditions for the highest removal efficiency of petroleum oil and other contaminants in the refinery wastewater.

### Treatment of domestic wastewater using the unmodified AS reactor (1st trial)

#### Activated sludge culture operation conditions

Characterization of the AS culture (Table 2A) in the developed unmodified reactor recorded DO levels ranging between 3.2 and 4.7 mg/L during the domestic wastewater treatment trial period (12 days) which revealed a good amount of oxygen in the aeration basin. pH recorded very close levels along the treatment period (7.1–7.6), indicating mostly stable slight alkaline conditions. SV_30_ ranged between a minimum of 120 mL/L at the 1st treatment day that was gradually increased to reach a maximum of 152 mL/L at the end of the duration period indicating reasonable SS content and good settleable solid conditions.

**Table 2:**
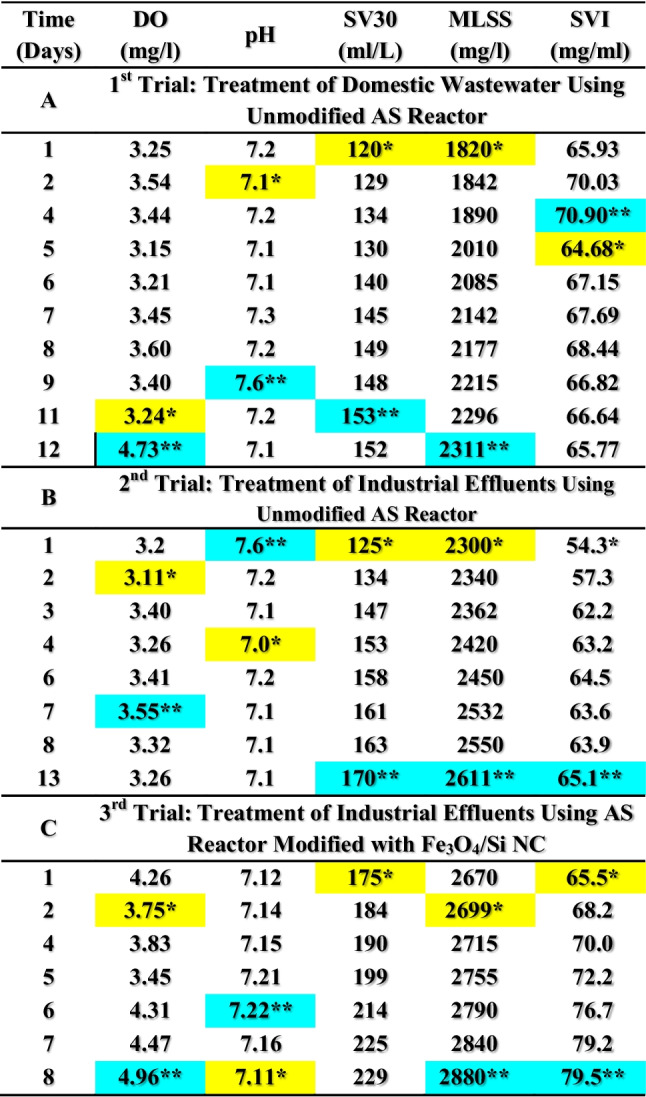
Activated sludge characterization during the treatability trials^@^

^@^Values with an average of 3 readings

^**^The highest value

^*^The lowest value

During this stage, high MLSS averages were determined with a clear regular trend of increasing MLSS content with time (1820 and 2311 mg/L at the 1st and last treatment days, respectively), indicating healthy sludge conditions. SVI that reflects sludge characters showed a stable behavior with values ranging between 64.7 and 70.9 mL/mg indicating good settleability and compatibility properties of the tested sludge culture.

### Treatability of raw domestic wastewater

The detailed levels of the pollutants load in the raw domestic wastewater, their highest and lowest REs%, their RCs, and their MPLs according to the Egyptian environmental regulation law (No. 48/ 1982 Clause 52 Modified by Decree 92/2013) for safe discharge into the marine environment are presented in Supplementary table (S8) and summarized in Table 3. Raw domestic wastewater contained high levels of TSS, TDS, BOD, COD, and OG. Levels of temperature, pH, and DO in the treated effluents complied with the Egyptian environmental regulation law for safe discharge into aquatic environments (non-freshwater).

**Table 3:** Highest and lowest REs% of the different parameters in the raw and treated domestic wastewater during treatment using the unmodified AS reactor

Parameter	Raw wastewater (mg/L)	Highest value/RE%	RC (mg/L)	Lowest value/RE%	MPL (mg/L) ^a^
Temperature	20.5 ± 0.42	21.5 ± 0.28	-----	19.7 ± 0.37	< 3 ºC*
pH	7.2 ± 0.57	7.6 ± 0.63	-----	7.1 ± 0.57	6–9
DO	3.5 ± 0.72	5.81 ± 0.92	-----	4.45 ± 0.26	˃ 4
TDS	3000 ± 0.34	6.27 ± 0.41	2812 ± 0.41	0.87 ± 0.895	2000
TSS	280 ± 0.09	61.1 ± 0.95	109 ± 0.95	37.5 ± 0.37	50
BOD	1600 ± 0.66	64.10 ± 0.99	575 ± 0.99	10.94 ± 0.34	60
COD	2900 ± 0.37	62.1 ± 0.86	1100 ± 0.86	17.24 ± 0.69	80
OG	215 ± 0.43	57.21 ± 0.29	92 ± 0.29	5.12 ± 0.28	10

^*^Higher than the receiving water temperature

^a^Egyptian environmental regulation law (No. 48/ 1982 Clause 52 Modified by Decree 92/2013)

**Fig. 4:**
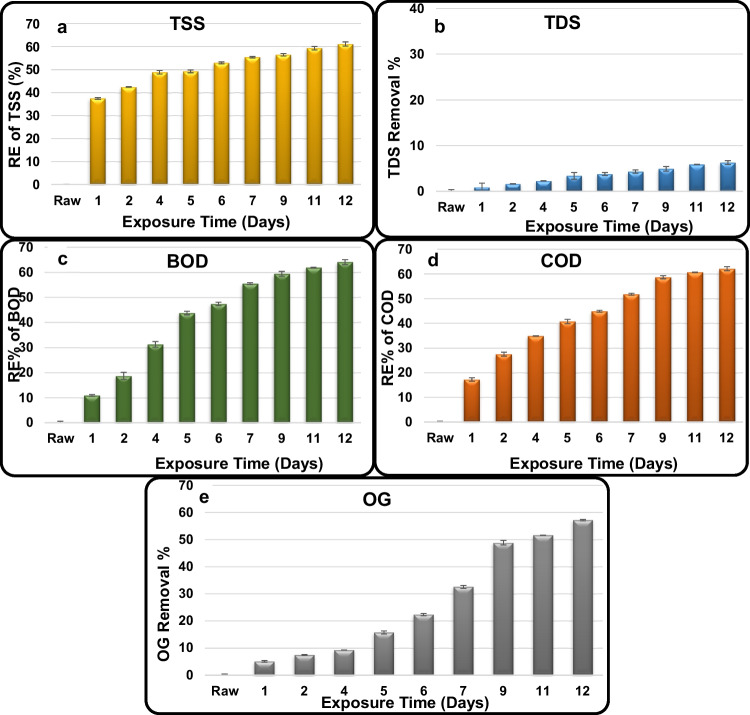
Removal efficiency % of **a** TSS, **b** TDS, **c** BOD, **d** COD, and **e** OG in the domestic wastewater treated using unmodified AS reactor

TSS, TDS, BOD, COD, and OG exhibited a very clear trend of decreasing their RCs with increasing exposure time reaching their highest REs% and their lowest concentrations at the last tested exposure (12 days). The highest RE of TSS from raw domestic wastewater recorded 61.1 ± 0.9% (Fig. 4a), equivalent to 109 ± 0.9 mg/L, which is 2.2-fold higher than the MPL of TSS (50 mg/L). The highest RE of TDS from raw domestic wastewater recorded 6.3 ± 0.4% (Fig. 4b), equivalent to 2812 ± 0.4 mg/L, which is 1.4-fold higher than the MPL of TDS (2000 mg/L, domestic effluents). The highest RE of BOD from raw domestic wastewater recorded 64.1 ± 0.99% (Fig. 4c), equivalent to 575 ± 0.99 mg/L, which is 9.6-fold higher than the MPL of BOD (60 mg/L). The highest RE of COD from raw domestic wastewater recorded 62.1 ± 0.9% (Fig. 4d), equivalent to 1100 ± 0.9 mg/L, which is 13.8-fold higher than the MPL of the COD (80 mg/L). The highest RE of OG from raw domestic wastewater recorded 57.2 ± 0.3% (Fig. 4e), equivalent to 92 ± 0.3 mg/L, which is 9.2-fold higher than the MPL of OG (10 mg/L).

The remarkably lower REs of TDS (0.87 ± 0.895–6.27 ± 0.41%) compared to those achieved for the TSS is mainly attributed to biological treatment that is well known to increase TDS levels due to the decomposition of complex organic matter into soluble salts. Similarly, none of the TSS, BOD, COD, and OG could reach safe levels in the effluent treated using the unmodified AS reactor. This is mainly attributed to, first, their high initial levels in the raw domestic wastewater (280 ± 0.1, 1600 ± 0.7, 2900 ± 0.4, and 215 ± 0.4 mg/L, respectively) and, second, to the fact that AS treatment received the raw domestic influent without any preliminary or primary treatment (sedimentation) that is known to remove up to 35% organic matter included in the wastewater, which in turn enhanced their removal. Also, the absence of physical treatment has led to a reduction in OG removal, which is normally floated on the wastewater surface and usually removed physically during the primary treatment.

As shown above, treatment of domestic wastewater using unmodified AS system achieved 61.1, 6.27, 64.10, 62.1, and 57.21% as the highest removals for TSS, TDS, BOD, COD, and OG (109, 2812, 575, 1100, and 920.29 mg/L), respectively, all after 12 days. These residues are 2.18-, 1.41-, 9.58-, 13.75-, and 9.2-fold higher than their MPLs of 50, 2000, 60, 80, and 10 mg/L, respectively, stated by the Egyptian laws. The achieved results are highly supported by a recent similar study of domestic wastewater purification in two household wastewater treatment plants (WWTPs) (Poland). The average removal efficiencies of BOD_5_ and COD were 66–83 and 62–65%, respectively. Much lower effects were obtained for TSS (17–48%), total phosphorus (TP), and total nitrogen (TN) removal, which did not exceed 34%. Thus, in the case of biogenic compounds, the analyzed systems did not guarantee that the quality of treated wastewater would meet the requirements of the Polish law during any period of operation (Karczmarczyk et al. 2021, Micek et al. 2021). Other workers clearly demonstrated that there will always be a group of compounds poorly removed/eliminated using typical conventional biological (secondary) treatment such as activated sludge (Grandclement et al. 2017, Tran et al. 2018). Therefore, modification with advanced treatment is necessary (Yang et al. 2017). As suggested in the present study, modification of AS reactor using new Fe_3_O_4_/silica NC to produce high-quality treated effluents for potential reuse, others suggested that activated carbon adsorption, either as powdered (PAC) or granular (GAC) forms, is considered an efficient and cost-efficient technology for controlling organic micro contaminants (Campinas et al. 2022, Rizzo et al. 2019, Helmreich and Metzger 2017). They stated that PAC dosing directly into a conventional AS reactor (PAC-AS) has the advantage of ensuring PAC exhaustion (very long PAC residence time, equal to the sludge age) without requiring additional investment (Çeçen and Aktas 2012), nor increased energy consumption. Moreover, no extra pumping is required for PAC recirculation and PAC particles are separated from the treated water together with the excess sludge.

### Treatment of industrial wastewater using the unmodified AS reactor (2nd trial)

#### Activated sludge culture operation conditions

Characterization of the AS culture (Table 2B) in the developed unmodified reactor recorded DO levels ranging between 3.1 and 3.6 mg/L in the AS basin during the treatment period (13 days), which appeared good amount of oxygen in the aeration basin. pH recorded very close levels along the treatment period (7.0–7.6), indicating mostly stable slight alkaline conditions. SV_30_ ranged between a minimum of 125 mL/L at the 1st treatment day that was gradually increased to reach a maximum of 170 mL/L at the end of the duration period (13 days), indicating reasonable SS content and good settleable solid conditions.

Higher MLSS averages were recorded during the second run indicating maturation of the culture compared with the first run. MLSS averages increased in a clear and regular trend with increasing time recording the minimum of 2300 mg/L at the 1st treatment day that was steadily increased to record the maximum value of 2611 mg/L at the last treatment day (12 days), indicating healthy sludge conditions. SVI showed stable behavior with values ranging between 54.3 and 65.1 mL/mg, indicating good settleability and compatibility properties of the tested sludge culture.

### Treatability of industrial wastewater

The detailed levels of the pollutants load in the industrial wastewater, their highest and lowest REs%, and RCs are presented in the Supplementary table (S9) and summarized in Table 4. Raw industrial oily wastewater contained high levels of TSS, TDS, BOD, COD, and OG (Table S9). Levels of temperature, pH, and DO in the treated effluents complied with the Egyptian environmental regulations for safe discharge into non–freshwater aquatic environments.

**Table 4:** Highest and lowest REs% of the different parameters in the raw and treated Industrial oily wastewater during treatment using the unmodified AS reactor

Parameter	Raw wastewater (mg/l)	Highest value/RE%	RC (mg/l)	Lowest value/RE%	MPL (mg/l) ^a^
Temperature	20.8 ± 0.63	21.7 ± 0.66	-----	20.0 ± 0.12	< 3 ºC*
pH	7.21 ± 0.32	7.24 ± 0.99	-----	7.11 ± 0.01	6–9
DO	4.8 ± 0.26	5.6 ± 0.66	-----	4.4 ± 0.61	˃ 4
TSS	300 ± 0.38	55.3 ± 0.35	134 ± 0.35	16.7 ± 0.65	50
TDS	3100 ± 0.34	12.6 ± 0.34	2710 ± 0.34	0.81 ± 0.75	5000
BOD	1200 ± 0.38	50.0 ± 0.07	600 ± 0.07	6.67 ± 0.36	60
COD	2342 ± 0.57	40.22 ± 0.17	1400 ± 0.17	5.64 ± 0.89	80
OG	380 ± 0.61	56.84 ± 0.36	164 ± 0.36	10.53 ± 0.87	10

^*^Higher than the receiving water temperature

^a^Egyptian environmental regulation law (No. 48/ 1982 Clause 52 Modified by Decree 92/2013)

**Fig. 5:**
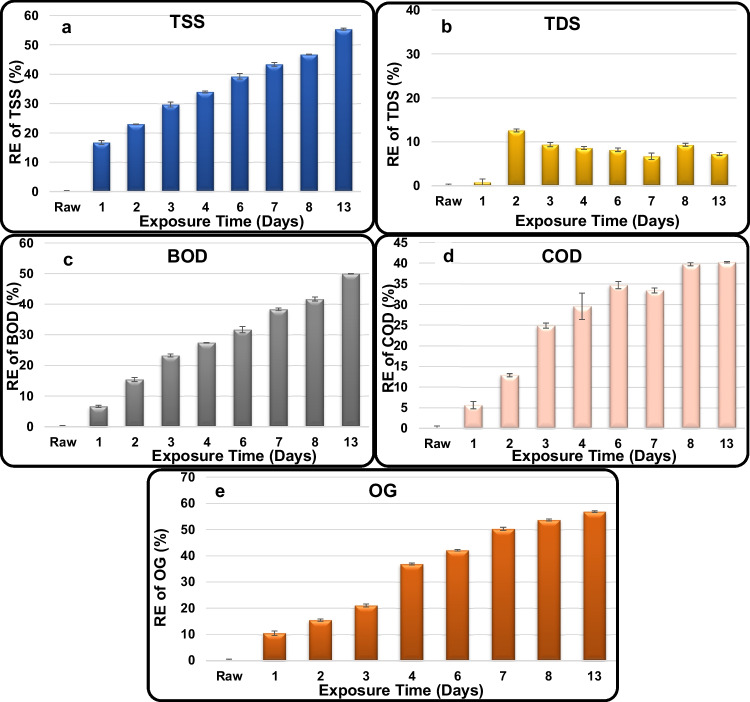
Removal efficiency % of **a** TSS, **b** TDS, **c** BOD, **d** COD, and **e** OG in the industrial wastewater treated using unmodified AS reactor

TSS, BOD, COD, and OG exhibited a very clear trend of decreasing their RCs with increasing exposure time reaching the lowest concentration at the last tested exposure (13 days). The highest RE of TSS recorded 55.3 ± 0.4%, equivalent to 134 ± 0.4 mg/L, which is 2.7-fold higher than its MPL (50 mg/L) (Fig. 5a). The highest RE of TDS was recorded at 12.6 ± 0.3%, equivalent to 2710 ± 0.3 mg/L, which is much below its MPL (5000 mg/L, industrial effluents) (Fig. 5b). The highest RE of BOD recorded 50.0 ± 0.1%, equivalent to 600 ± 0.1 mg/L, which is 10.0-fold higher than its MPL (60 mg/L) (Fig. 5c). The highest RE of COD recorded 40.2 ± 0.2%, equivalent to 1400 ± 0.2 mg/L, which is 17.5-fold higher than its MPL (80 mg/L) (Fig. 5d). The highest RE of OG s recorded 56.8 ± 0.4%, equivalent to 164 ± 0.4 mg/L, which is 16.4-fold higher than its MPL (10 mg/L) (Fig. 5e).

REs of TDS are remarkably lower than TSS removal, and none of the TSS, BOD, COD, and OG could reach safe levels in the effluent treated using the unmodified AS reactor. Moreover, REs achieved (55.3, 12.6, 50.0, 40.22, and 56.84%, respectively) after 12 days were lower than those obtained for domestic wastewater. This is mainly attributed to the toxicity of oil hydrocarbons included in the industrial wastewater on the reactor microorganisms; the high initial levels of TSS, BOD, COD, and OG in the raw oily industrial wastewater; and the absence of preliminary or primary treatment (sedimentation). Other researchers reported that oil refineries produce contaminated wastewater that is highly inhibitory and toxic to the microbial activity of the AS system and makes wastewater treatment in the oil refinery unit even more challenging (Kalash and Albayati 2021). To overcome such drawbacks and low efficiency of the traditional AS of oil refinery wastewater, new more efficient treatment systems are proposed for producing a high-quality effluent, which can be reused in several industrial applications (Fiorenza et al. 2020; Ajith et al. 2021; Kamali et al. 2019).

### Treatment of industrial wastewater using Fe_3_O_4_/silica NC–modified AS system (3rd trial)

#### Activated sludge culture operation conditions

Characterization of the AS culture (Table 2C) in the developed Fe_3_O_4_/silica NC–modified reactor during the treatment of industrial wastewater from an oil refinery recorded DO levels ranged between 3.5 and 4.96 mg/L in the AS basin during the treatment period (8 days), which appeared good amount of oxygen in the aeration basin. pH recorded very close levels (7.1–7.2) along the treatment period indicating mostly stable slight alkaline conditions. SV_30_ ranged between a minimum of 175 mL/L at the 1st treatment day that was gradually increased to reach a maximum of 229 mL/L at the end of the duration period indicating reasonable SS content and good settleable solid conditions.

Higher MLSS averages were recorded during the third run indicating maturation of the culture compared with the first two runs. MLSS averages increased in a clear and regular trend with increasing time recording the minimum of 2670 mg/L on the 1st treatment day that was steadily increased to record the maximum value of 2880 mg/L on the 8th treatment day, indicating healthy sludge conditions. SVI showed stable behavior with values ranging between 65.5 and 79.5 mL/mg, indicating good settleability and compatibility properties of the tested sludge culture.

### Treatability of industrial oily wastewater

The detailed levels of the pollutants load in the industrial wastewater, their highest and lowest REs%, and RCs are presented in the Supplementary table (S10) and summarized in Table 5. Raw industrial oily wastewater contained much higher levels of temperature, BOD, COD, and OG as well as lower levels of DO, pH, and TDS (S10) compared to those in the second run. Levels of temperature, pH, and DO in the treated effluents complied with the Egyptian environmental regulations for safe discharge into non–freshwater aquatic environments.

**Table 5:** Highest and lowest REs% of the different parameters in the raw and treated industrial oily wastewater during treatment using the Fe_3_O_4_/silica NC–modified AS reactor

Parameter	Raw wastewater (mg/l)	Highest value/RE%	RC (mg/l)	Lowest value/RE%	MPL (mg/l)^a^
Temperature	25.1 ± 0.61	26.1 ± 0.71	-----	24 ± 0.93	< 3 ºC*
pH	6.98 ± 0.42	7.14 ± 0.34	-----	7.0 ± 0.43	6–9
DO	4.1 ± 0.44	4.96 ± 0.98	-----	3.45 ± 0.75	˃ 4
TSS	300 ± 0.57	78.33 ± 0.36	65 ± 0.36	16.67 ± 0.66	50
TDS	2500 ± 0.89	3.6 ± 0.87	2410 ± 0.87	0 ± 0.89	5000
BOD	1700 ± 0.37	87.65 ± 0.44	210 ± 0.44	21.18 ± 0.43	60
COD	2900 ± 0.41	85.17 ± 0.44	430 ± 0.44	17.24 ± 0.57	80
OG	480 ± 0.34	92.92 ± 0.84	34 ± 0.84	24.79 ± 0.03	10

^*^Higher than the receiving water temperature

^a^Egyptian environmental regulation law (No. 48/ 1982 Clause 52 Modified by Decree 92/2013)

**Fig. 6:**
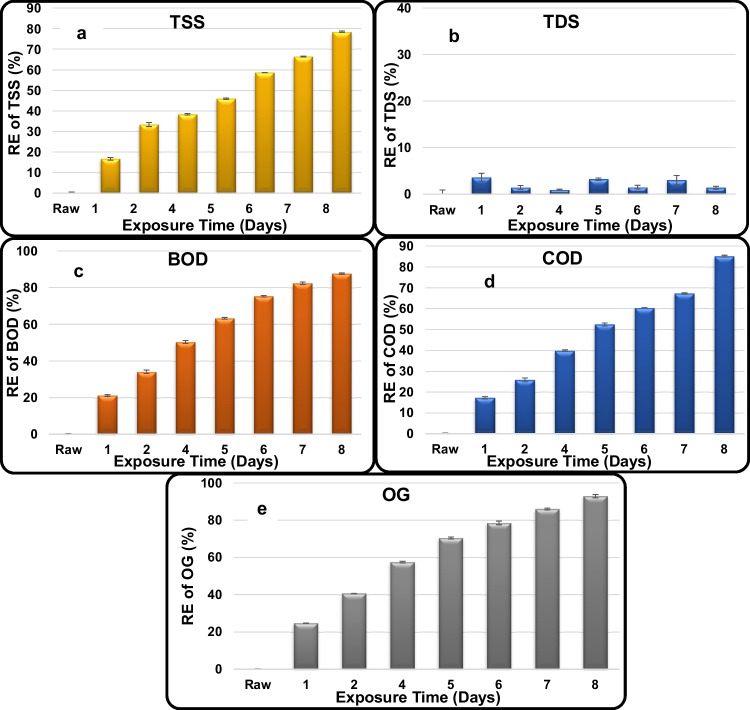
Removal efficiency % of **a** TSS, **b** TDS, **c** BOD, **d** COD, and **e** OG in the industrial wastewater treated using Fe_3_O_4_/silica nanocomposite modified AS reactor

TSS, BOD, COD, and OG exhibited a very clear trend of decreasing their RCs with increasing exposure time reaching the lowest concentration at the last tested exposure (8 days). As a general fact, modification of the AS system using Fe_3_O_4_/silica NC remarkably enhanced REs of the included contaminants and produce relatively high-quality effluents although much higher initial concentrations are present. The highest RE of TSS recorded 78.3 ± 0.4%, equivalent to 65 ± 0.4 mg/L, which is 1.3-fold higher than the its MPL (50 mg/L) (Fig. 6a). The highest RE of TDS recorded 3.6 ± 0.9%, equivalent to 2410 ± 0.9 mg/L, which is much below its MPL (5000 mg/L) (Fig. 6b). The highest RE of BOD recorded 87.7 ± 0.4%, equivalent to 210 ± 0.4 mg/L, which is 3.5-fold higher than its MPL (60 mg/L) (Fig. 6c). The highest RE of COD recorded 85.2 ± 0.4%, equivalent to 430 ± 0.4 mg/L, which is 5.4-fold higher than its MPL (80 mg/L) (Fig. 6d). The highest RE of OG recorded 92.9 ± 0.8%, equivalent to 34 ± 0.8 mg/L, which is 3.4-fold higher than its MPL (10 mg/L) (Fig. 6e).

The remarkably increased REs of the industrial contaminants are mainly attributed to the modification of the AS system with the Fe_3_O_4_/silica NC. However, TSS, BOD, COD, and OG are still slightly above their MPLs. This can be overcome either by increasing the hydraulic retention time in the aeration tank or by applying preliminary or primary treatment (sedimentation) before the biological (AS) treatment.

In the present study, magnetite nanoparticles (Fe_3_O_4_ NPs) and magnetite silica (Fe_3_O_4_/silica) nanocomposites (NCs) were used to modify and enhance AS system efficiency and performance to treat oil refinery wastewater compared to their treatment using unmodified AS system. Modification of AS system with Fe_3_O_4_/silica NC remarkably enhanced the REs of all the included contaminants and produce relatively high-quality effluents although much higher initial concentrations were present. The highest REs are 78.33, 3.6, 87.65, 85.17, and 92.92% of TSS, TDS, BOD, COD, and OG, respectively, all after 8 days. Levels of temperature, pH, and DO in the treated effluents complied with the Egyptian environmental law while TSS, BOD, COD, and OG were still slightly above their MPLs. However, their removal can be optimized by changing operation conditions such as increasing hydraulic retention time and nanomaterial doses and/or applying the primary treatment stage before biological treatment. Recently, many studies were conducted using nanomaterials for the purification of water/wastewater either on their own or to modify other traditional treatment processes as in the present study, and prove high efficiency and remarkably optimize the process. Examples include remediation of oil refinery wastewater using functionalized mesoporous materials MCM-41 in batch and continuous adsorption process (Kalash and Albayati 2021), zero-valent iron nanoparticles (nZVI) for the efficient catalytic treatment of high-strength domestic wastewater (Ansari et al. 2022), carbon nanotubes and nanomaterials in wastewater treatment applications (Kamali et al. 2019; Rice et al. 2017; Almusawy et al. 2020; Abdelbasir and Shalan 2019), and many others.

Table 6 compares between the unmodified and Fe_3_O_4_/silica NC–modified AS reactors towards the removal of the industrial contaminants. The following points are concluded:

1. It is very clear that the lowest REs of all the tested parameters were achieved by the unmodified AS treatment.
2. Integration of the AS treatment with Fe_3_O_4_/silica NC remarkably enhanced the REs of all the tested parameters compared to NC-free AS treatment (AS control).
3. TSS removal increased from 55.3% (control) to 78.3% (Fe_3_O_4_/silica NC) reaching 65 mg/L, slightly above its MPL (50 mg/l).
4. TDS recorded 12.6% as the highest RE when treated with the control that decreased to 3.6% after treatment with Fe_3_O_4_/silica NC and highly complied with its MPL (5000 mg/L). For TDS, the efficiency of the treatment was inversely related to its RC, i.e., increasing TDS levels indicate efficient treatment, which confirms the active decomposition of organic matter accompanied by the formation of soluble salts.
5. The highest REs achieved for BOD recorded 50.0 and 87.7%, equivalent to an RC_F_ of 600 and 210 mg/L in the final effluent treated with the AS control and Fe_3_O_4_/silica NC, respectively, confirming the positive effect of the NC.
6. Similarly, the highest REs achieved for COD recorded 40.2 and 85.2% (RC_F_: 1400 and 430 mg/L) after treatment using AS control and Fe_3_O_4_/silica NC, respectively.
7. Finally, the highest REs archived for OG recorded 56.5 and 92.9% (RC_F_: 164 and 34 mg/L) by the two technologies, respectively.

**Table 6:** Comparison of the removal efficiency of industrial contaminants by the different applied treatment technologies

Treatment technology	The highest RE/RC_I_ and RC_F_ (mg/L) **				
	TSS	TDS	BOD	COD	OG
Unmodified AS technology	55.3 ± 0.35% RC_I_: 300 ± 0.38 RC_F_: 134 ± 0.35	12.6 ± 0.34% RC_I_: 3100 ± 0.34 RC_F_: 2710 ± 0.34	50.0 ± 0.07% RC_I_: 1200 ± 0.38 RC_F_: 600 ± 0.07	40.22 ± 0.17% RC_I_: 2342 ± 0.57 RC_F_: 1400 ± 0.17	56.84 ± 0.36% RC_I_: 380 ± 0.61 RC_F_: 164 ± 0.36
Fe_3_O_4_/silica nanocomposite–modified AS technology	78.33 ± 0.36% RC_I_: 300 ± 0.57 RC_F_: 65 ± 0.36	3.6 ± 0.87% RC_I_: 2500 ± 0.89 RC_F_: 2410 ± 0.87	87.65 ± 0.44% RC_I_: 1700 ± 0.37 RC_F_: 210 ± 0.44	85.17 ± 0.44% RC_I_: 2900 ± 0.41 RC_F_: 430 ± 0.44	92.92 ± 0.84% RC_I_: 480 ± 0.34 RC_F_: 34 ± 0.84
MPL (mg/l) ^a^	50	5000	60	80	10

^**^RC_I_, initial residual concentration; RC_F_, final residual concentration

^a^Egyptian environmental regulation law (No. 48/ 1982 Clause 52 Modified by Decree 92/2013)

## Conclusions

It is clear that the integration of the AS treatment with Fe_3_O_4_/silica NC is highly effective, promising, and economic for the treatment of highly toxic industrial wastewater such as petroleum refinery effluents. The results concluded the following points:

1. Fe_3_O_4_/silica NC–modified AS treatment produces high-quality effluents in a very short time (2 h) although much higher initial concentrations were present.
2. Concerning levels of some parameters that are still slightly higher than their MPLs, this can be overcome by increasing the hydraulic retention time in the aeration tank, increasing NC dosage, or applying preliminary or primary treatment (sedimentation) before the biological (AS) treatment.
3. Using the proposed NC-modified AS system reduced the treatment time and sludge produced during the process.
4. Finally, the proposed system represents a very promising, renewable, and cheap biotechnology for the treatment of wide range of contaminated effluents not only in the industrial sector but also for domestic and agricultural wastewater.

### Supplementary Information

Below is the link to the electronic supplementary material.

Supplementary file1 (DOCX 2371 KB)

Supplementary file2 (DOCX 97 KB)

## Data Availability

The authors confirm that the manuscript was not submitted to a preprint server before submitting it to *Environmental Science and Pollution Research* and data are not shared with any other party.
