# Supplementary material for: Effective treatment of petroleum oil–contaminated wastewater using activated sludge modified with magnetite/silicon nanocomposite
Source: Environ Sci Pollut Res Int. 2023 May 1;31(12):17634–50. doi: 10.1007/s11356-023-26557-6 (PMC11289328; doi:10.1007/s11356-023-26557-6)
Supplement: Supplementary file 1 — Supplementary file1 (DOCX 2371 KB) [file 11356_2023_26557_MOESM1_ESM.docx]

**
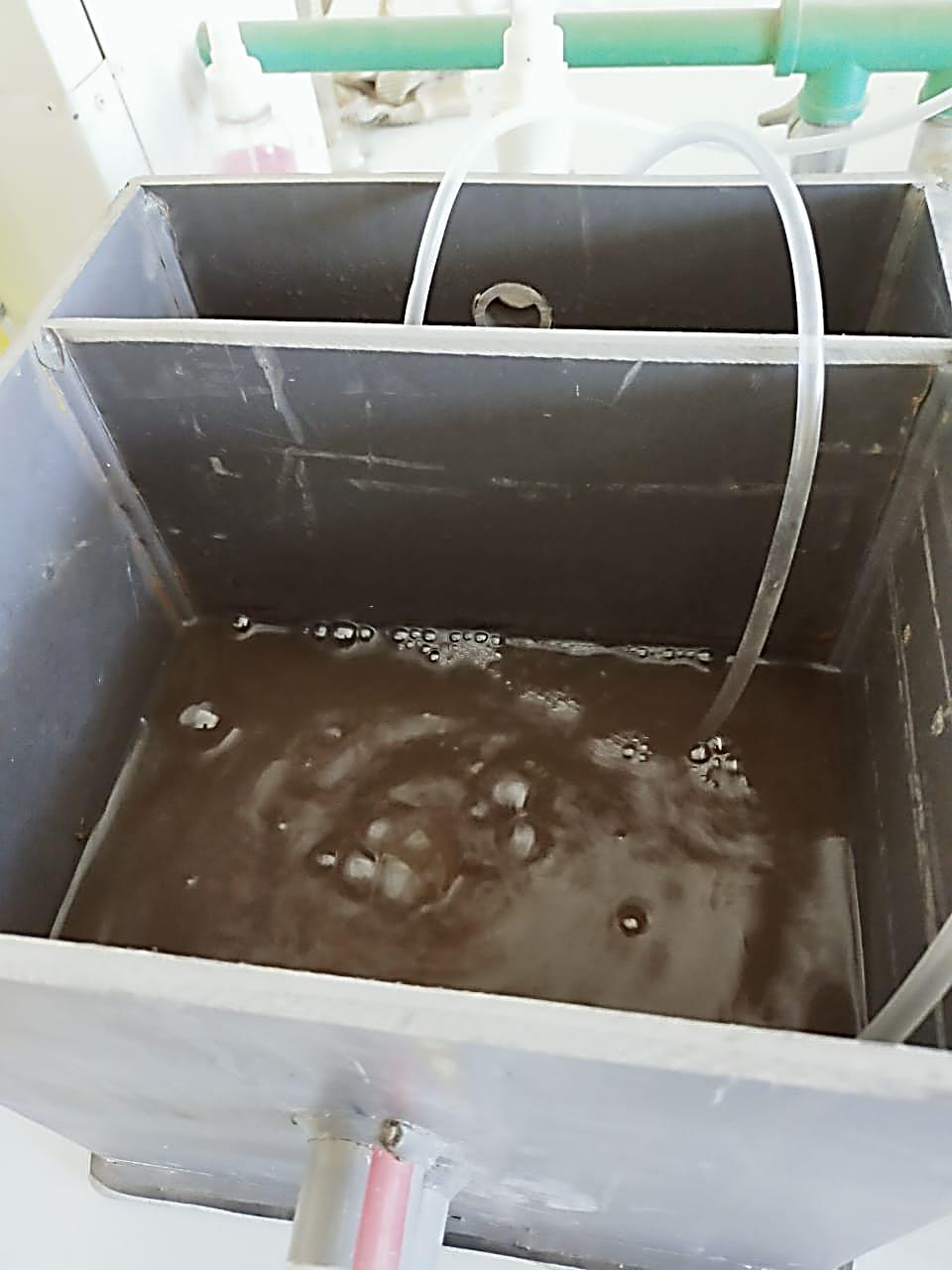

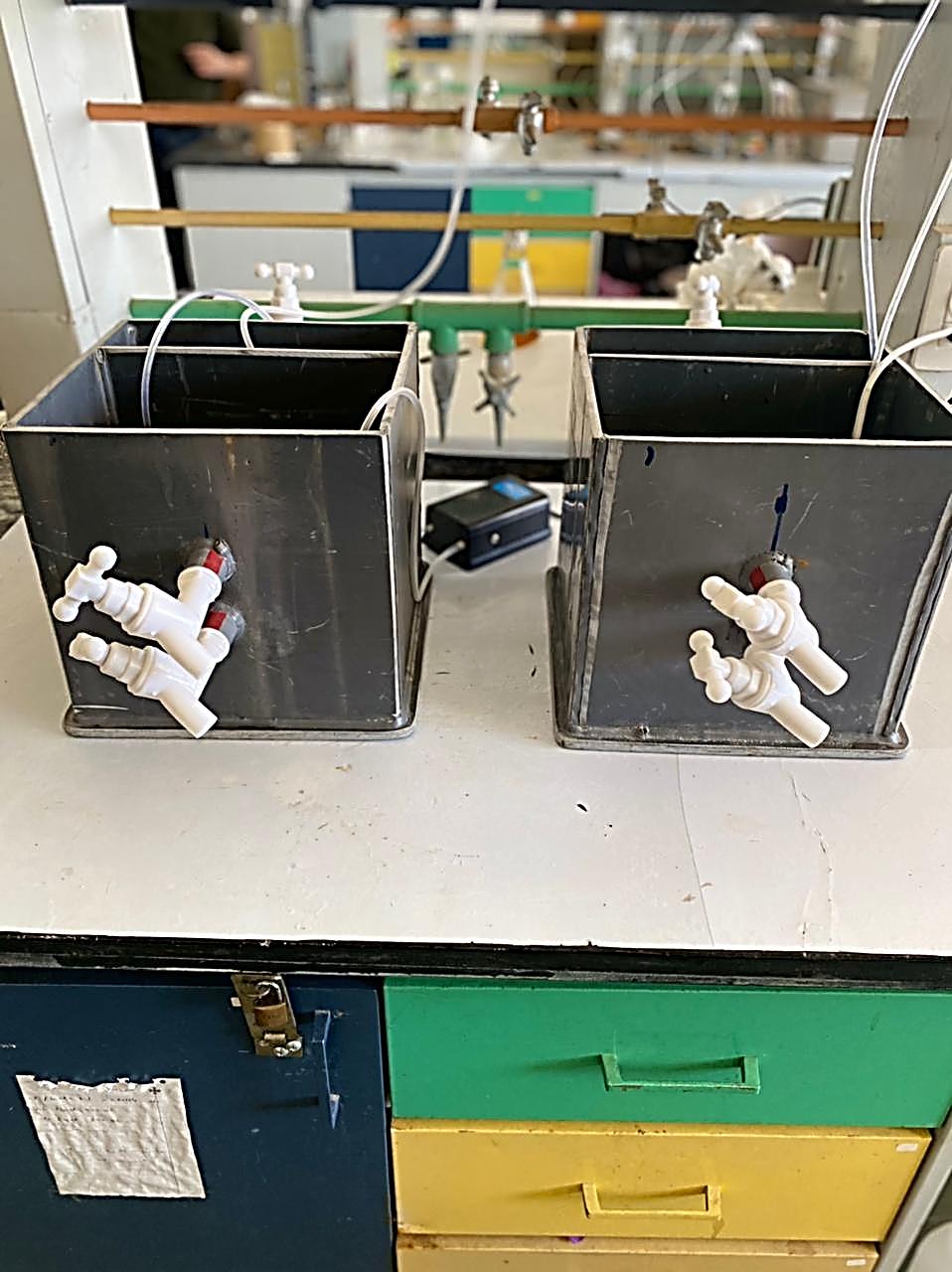
Supplementary Materials**

**S 1: Bench Scale AS Unit**

**
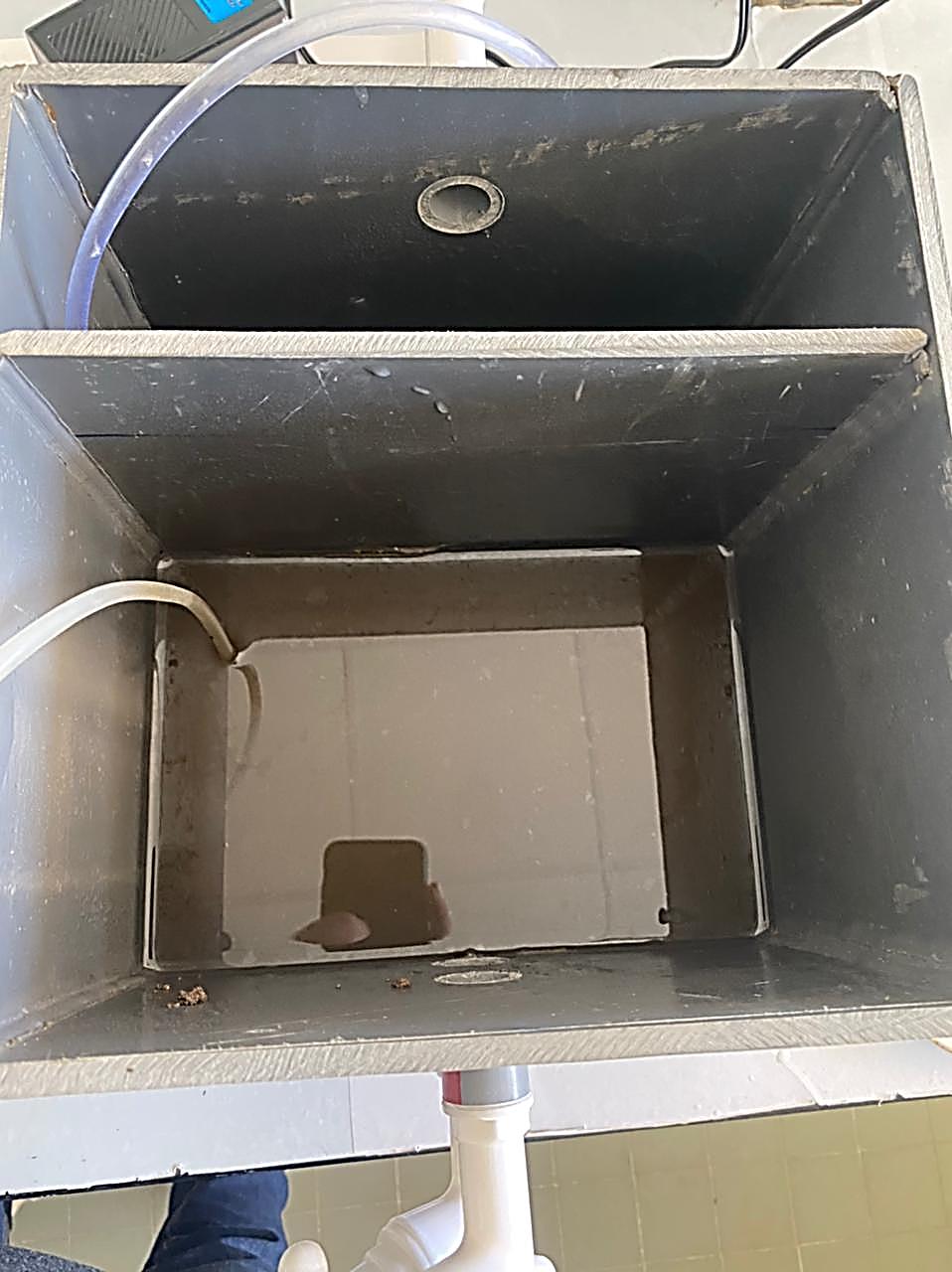
 S 1: Top View of the AS Unit**

**
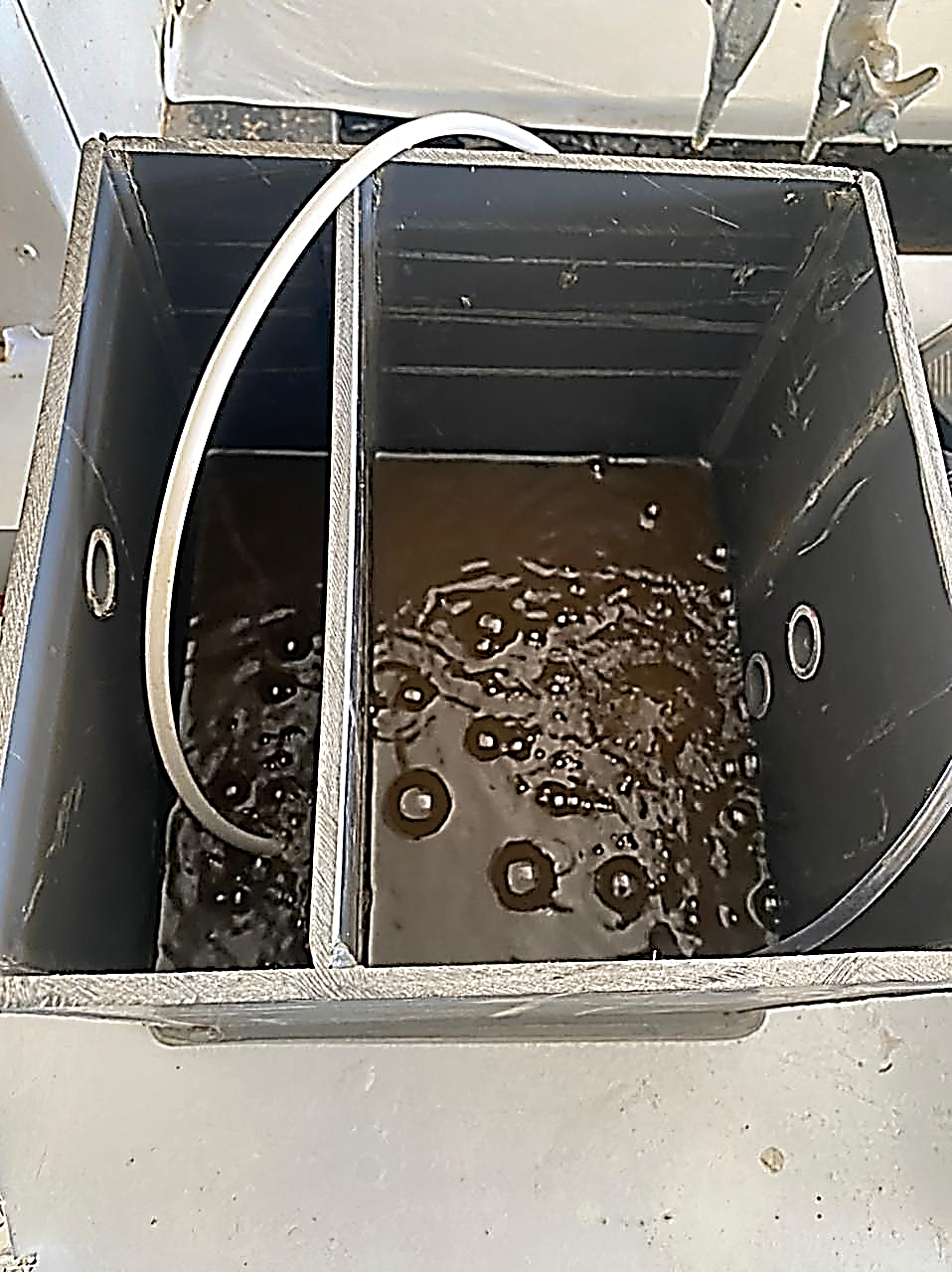

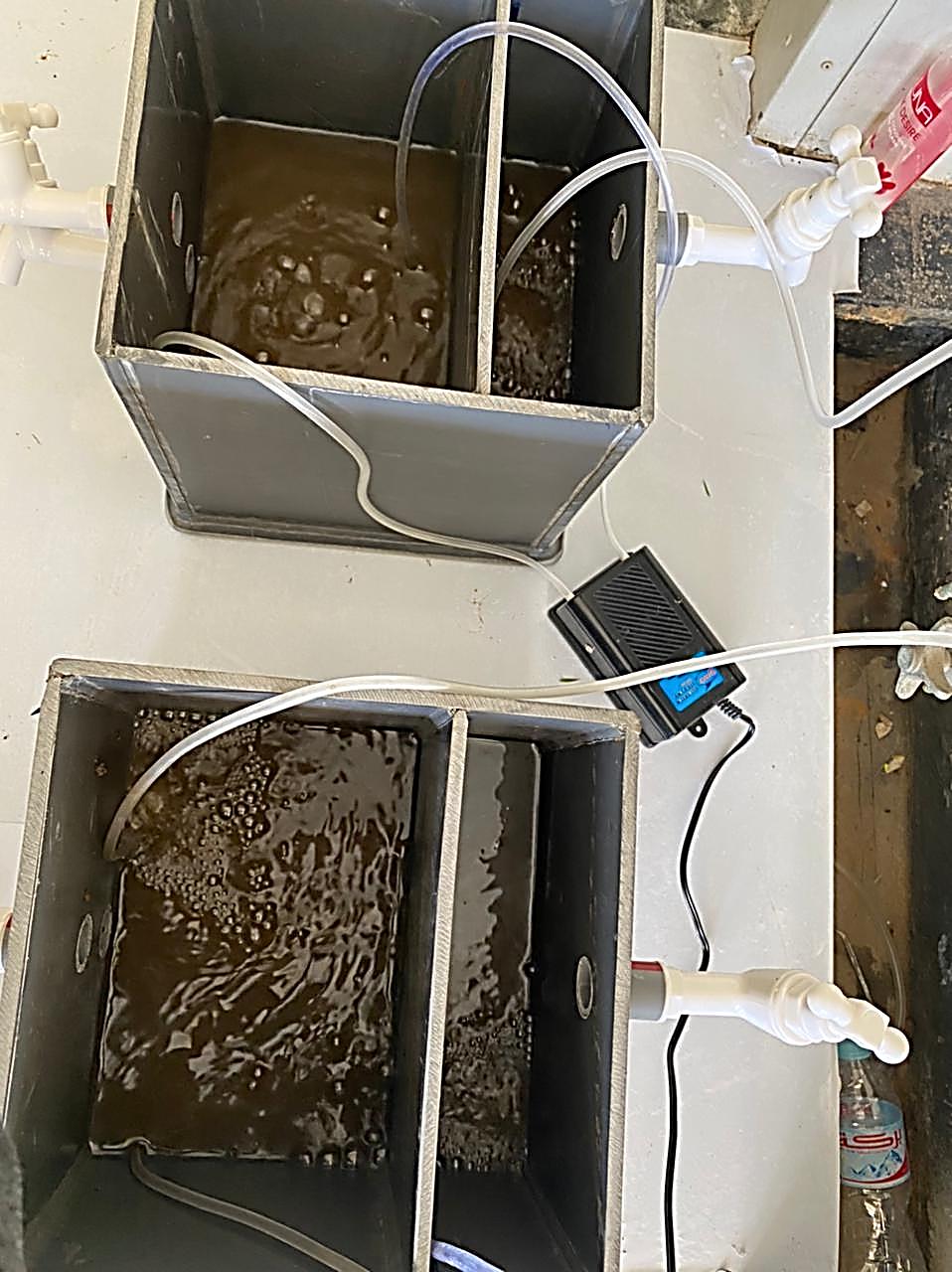
S 3: Aeration of the Activated Sludge (AS) Culture during Maturation Process**
