# Supplementary material for: Effective treatment of petroleum oil–contaminated wastewater using activated sludge modified with magnetite/silicon nanocomposite
Source: Environ Sci Pollut Res Int. 2023 May 1;31(12):17634–50. doi: 10.1007/s11356-023-26557-6 (PMC11289328; doi:10.1007/s11356-023-26557-6)
Supplement: Supplementary file 2 — Supplementary file2 (DOCX 97 KB) [file 11356_2023_26557_MOESM2_ESM.docx]

**(S 4)**

**Appendix 1: Residual Concentration (RC, mg/l) and Removal Efficiency (RE %) of Oil & Grease of AS-Treated Effluents**

| **Oct. 20** | **Oil RC**  **(mg/l)** | | **RE %** | **Nov. 20** | **Oil RC (mg/l)** | | **RE %** | **Dec. 20** | **Oil RC**  **(mg/l)** | | **RE %** | **Jan. 21** | **Oil RC**  **(mg/l)** | | **RE %** | **Feb. 21** | **Oil RC**  **(mg/l)** | | **RE %** | **Mar. 21** | **Oil RC**  **(mg/l)** | | **RE %** |
| --- | --- | --- | --- | --- | --- | --- | --- | --- | --- | --- | --- | --- | --- | --- | --- | --- | --- | --- | --- | --- | --- | --- | --- |
|  | **Infl.** | **Effl.** |  |  | **Infl.** | **Effl.** |  |  | **Infl.** | **Effl.** |  |  | **Infl.** | **Effl.** |  |  | **Infl.** | **Effl.** |  |  | **Infl.** | **Effl.** |  |
| **1** | **330** | **125** | **60** | **1** | **400** | **151** | **62.3** | **1** | **530** | **330** | **40** | **2** | **229** | **162** | **30** | **1** | **460** | **240** | **50** | **1** | **250** | **312** | **-20** |
| **2** | **450** | **134** | **70** | **2** | **500** | **152** | **69.6** | **2** | **191** | **241** | **-30** | **3** | **460** | **140** | **70** | **2** | **380** | **100** | **70** | **2** | **300** | **243** | **20** |
| **3** | **390** | **140** | **60** | **3** | **430** | **153** | **64.4** | **3** | **284** | **84** | **70** | **4** | **480** | **214** | **60** | **3** | **260** | **95** | **60** | **3** | **340** | **210** | **40** |
| **6** | **365** | **147** | **60** | **4** | **370** | **154** | **58.4** | **4** | **280** | **80** | **70** | **5** | **430** | **240** | **40** | **4** | **510** | **86** | **80** | **4** | **320** | **170** | **50** |
| **7** | **413** | **142** | **70** | **7** | **309** | **157** | **49.2** | **5** | **590** | **390** | **30** | **6** | **315** | **216** | **30** | **5** | **260** | **134** | **50** | **5** | **251** | **181** | **30** |
| **8** | **410** | **139** | **70** | **8** | **350** | **158** | **54.9** | **6** | **130** | **145** | **-10** | **7** | **310** | **200** | **40** | **6** | **150** | **127** | **20** | **6** | **420** | **173** | **60** |
| **9** | **200** | **124** | **40** | **9** | **370** | **159** | **57.0** | **9** | **440** | **240** | **50** | **10** | **316** | **219** | **30** | **7** | **110** | **77** | **30** | **7** | **320** | **161** | **50** |
| **10** | **300** | **132** | **60** | **10** | **230** | **160** | **30.4** | **10** | **487** | **287** | **40** | **11** | **330** | **177** | **50** | **8** | **310** | **64** | **80** | **8** | **347** | **120** | **70** |
| **11** | **360** | **108** | **70** | **11** | **310** | **161** | **48.1** | **11** | **455** | **255** | **40** | **12** | **310** | **189** | **40** | **9** | **280** | **124** | **60** | **9** | **396** | **143** | **60** |
| **13** | **390** | **188** | **50** | **12** | **520** | **162** | **68.8** | **12** | **440** | **240** | **50** | **13** | **265** | **180** | **30** | **10** | **580** | **171** | **70** | **10** | **380** | **140** | **60** |
| **14** | **430** | **172** | **60** | **15** | **380** | **165** | **56.6** | **13** | **480** | **280** | **40** | **14** | **340** | **165** | **50** | **11** | **325** | **140** | **60** | **11** | **386** | **145** | **60** |
| **15** | **410** | **177** | **60** | **16** | **280** | **166** | **40.7** | **14** | **410** | **210** | **50** | **15** | **320** | **151** | **50** | **12** | **318** | **200** | **40** | **12** | **310** | **132** | **60** |
| **16** | **390** | **169** | **60** | **17** | **310** | **167** | **46.1** | **17** | **270** | **70.0** | **70** | **18** | **480** | **160** | **70** | **13** | **210** | **185** | **10** | **13** | **295** | **120** | **60** |
| **17** | **380** | **158** | **60** | **18** | **320** | **168** | **47.5** | **18** | **294** | **94** | **70** | **19** | **150** | **246** | **-60** | **14** | **230** | **137** | **40** | **14** | **410** | **110** | **70** |
| **18** | **330** | **150** | **50** | **19** | **330** | **169** | **48.8** | **19** | **260** | **160** | **40** | **20** | **320** | **230** | **30** | **15** | **560** | **100** | **80** | **15** | **178** | **231** | **-30** |
| **19** | **290** | **125** | **60** | **20** | **600** | **170** | **71.7** | **20** | **480** | **280** | **40** | **21** | **330** | **209** | **40** | **16** | **480** | **263** | **50** | **16** | **380** | **200** | **50** |
| **22** | **293** | **113** | **60** | **23** | **242** | **173** | **28.5** | **21** | **320** | **120** | **60** | **22** | **300** | **200** | **30** | **17** | **580** | **200** | **70** | **17** | **500** | **184** | **60** |
| **23** | **320** | **142** | **60** | **24** | **230** | **174** | **24.3** | **22** | **140** | **60** | **60** | **23** | **330** | **210** | **40** | **18** | **580** | **230** | **60** | **18** | **180** | **243** | **-40** |
| **24** | **300** | **120** | **60** | **25** | **340** | **175** | **48.5** | **25** | **371** | **171** | **50** | **26** | **400** | **241** | **40** | **19** | **630** | **271** | **60** | **19** | **480** | **222** | **50** |
| **25** | **250** | **74** | **70** | **26** | **318** | **176** | **44.7** | **26** | **315** | **115** | **60** | **28** | **280** | **230** | **20** | **20** | **526** | **265** | **50** | **20** | **280** | **189** | **30** |
| **26** | **301** | **136** | **50** | **27** | **340** | **177** | **47.9** | **29** | **290** | **135** | **50** | **29** | **190** | **210** | **-10** | **21** | **250** | **274** | **-10** | **21** | **640** | **179** | **70** |
| **27** | **295** | **144** | **50** | **28** | **310** | **178** | **42.6** | **30** | **226** | **122** | **50** | **30** | **170** | **200** | **-20** | **22** | **1000** | **142** | **90** | **22** | **400** | **249** | **40** |
|  | | | | | | | | | | | | **31** | **480** | **192** | **60** | **23** | **1200** | **790** | **30** | **23** | **276** | **230** | **20** |
|  | | | | | | | | | | | | | | | | **24** | **480** | **600** | **-30** | **24** | **490** | **196** | **60** |
|  | | | | | | | | | | | | | | | | **25** | **640** | **700** | **-10** | **25** | **360** | **184** | **50** |
|  | | | | | | | | | | | | | | | | **26** | **465** | **640** | **-40** | **26** | **380** | **165** | **60** |
|  | | | | | | | | | | | | | | | | **27** | **1197** | **580** | **50** | **27** | **200** | **167** | **20** |
|  | | | | | | | | | | | | | | | | **28** | **276** | **400** | **-40** | **28** | **420** | **142** | **70** |
|  | | | | | | | | | | | | | | | | | | | | **29** | **370** | **140** | **60** |
|  | | | | | | | | | | | | | | | | | | | | **30** | **380** | **288** | **20** |
|  | | | | | | | | | | | | | | | | | | | | **31** | **370** | **273** | **30** |
| **Avg.** | **345.32** | **139.05** | **59.55** | **Avg.** | **354** | **164.8** | **50.5** | **Avg.** | **349.2** | **186.8** | **45** | **Avg.** | **332.1** | **200.9** | **33.2** | **Avg.** | **510.3** | **297.9** | **36.4** | **Avg.** | **366.6** | **187.7** | **42.3** |
| **Max.** | **450** | **188** | **70** | **Max.** | **600** | **178** | **71.7** | **Max.** | **590** | **390** | **70** | **Max.** | **480** | **246.0** | **70.0** | **Max.** | **1200** | **790.0** | **90** | **Max.** | **640.0** | **288.0** | **70** |
| **Min.** | **200** | **74.0** | **40** | **Min.** | **230** | **151** | **24.3** | **Min.** | **130** | **60.0** | **-30** | **Min.** | **150** | **140.0** | **-60.0** | **Min.** | **110.0** | **64.0** | **-40** | **Min.** | **178.0** | **110.0** | **-40** |
| **SD** | **62.01** | **24.63** | **7.67** | **SD** | **90.08** | **8.43** | **12.5** | **SD** | **124.2** | **91.1** | **23.7** | **SD** | **91.94** | **28.78** | **29.29** | **SD** | **290.5** | **213.9** | **38.74** | **SD** | **106.6** | **48.8357** | **29.2** |

**(S 5)**

**Appendix 2: Residual Concentration (RC, mg/l) and Removal Efficiency (RE %) of COD of AS-Treated Effluents**

| **Oct. 20** | **Oil RC**  **(mg/l)** | | **RE %** | **Nov. 20** | **Oil RC (mg/l)** | | **RE %** | **Dec. 20** | **Oil RC**  **(mg/l)** | | **RE %** | **Jan. 21** | **Oil RC**  **(mg/l)** | | **RE %** | **Feb. 21** | **Oil RC**  **(mg/l)** | | **RE %** | **Mar. 21** | **Oil RC**  **(mg/l)** | | **RE %** |
| --- | --- | --- | --- | --- | --- | --- | --- | --- | --- | --- | --- | --- | --- | --- | --- | --- | --- | --- | --- | --- | --- | --- | --- |
|  | **Infl.** | **Effl.** |  |  | **Infl.** | **Effl.** |  |  | **Infl.** | **Effl.** |  |  | **Infl.** | **Effl.** |  |  | **Infl.** | **Effl.** |  |  | **Infl.** | **Effl.** |  |
| **1** | **3000** | **1500** | **50.0** | **1** | **1720** | **988** | **42.56** | **1** | **2385** | **1155** | **50** | **2** | **1422** | **810** | **40** | **1** | **1150** | **840** | **30** | **1** | **1250** | **1092** | **10** |
| **2** | **3200** | **1420** | **55.63** | **2** | **1820** | **989** | **45.66** | **2** | **859.5** | **1100** | **-30** | **3** | **1980** | **970** | **50** | **2** | **950** | **350** | **60** | **2** | **1500** | **851** | **40** |
| **3** | **4000** | **1600** | **60.0** | **3** | **1750** | **990** | **43.43** | **3** | **1278** | **294** | **80** | **4** | **2000** | **1070** | **50** | **3** | **650** | **332.5** | **50** | **3** | **1700** | **735** | **60** |
| **6** | **2548** | **1274** | **50.0** | **4** | **1690** | **991** | **41.36** | **4** | **1260** | **280** | **80** | **5** | **1950** | **1200** | **40** | **4** | **1275** | **620** | **50** | **4** | **1600** | **595** | **60** |
| **7** | **3601** | **1455** | **59.59** | **7** | **1629** | **994** | **38.98** | **5** | **2655** | **1365** | **50** | **6** | **1835** | **1080** | **40** | **5** | **1150** | **469** | **60** | **5** | **1255** | **634** | **50** |
| **8** | **3520** | **1411** | **59.91** | **8** | **1670** | **995** | **40.42** | **6** | **585** | **507.5** | **10** | **7** | **1830** | **1000** | **50** | **6** | **900** | **444.5** | **50** | **6** | **2100** | **606** | **70** |
| **9** | **3300** | **1400** | **57.58** | **9** | **1690** | **996** | **41.07** | **9** | **1980** | **840** | **60** | **10** | **1836** | **1095** | **40** | **7** | **850** | **269.5** | **70** | **7** | **1600** | **564** | **60** |
| **10** | **3095** | **1366** | **55.86** | **10** | **1550** | **997** | **35.68** | **10** | **2191.5** | **1004.5** | **50** | **11** | **1850** | **885** | **50** | **8** | **775** | **224** | **70** | **8** | **1735** | **420** | **80** |
| **11** | **2041** | **1302** | **36.21** | **11** | **1630** | **998** | **38.77** | **11** | **2047.5** | **892.5** | **60** | **12** | **1830** | **945** | **50** | **9** | **700** | **432** | **40** | **9** | **1980** | **501** | **70** |
| **13** | **1850** | **1000** | **45.95** | **12** | **1840** | **999** | **45.71** | **12** | **1980** | **840** | **60** | **13** | **1785** | **900** | **50** | **10** | **1450** | **248.5** | **80** | **10** | **1900** | **490** | **70** |
| **14** | **3910** | **1458** | **62.71** | **15** | **1700** | **1002** | **41.06** | **13** | **2160** | **980** | **50** | **14** | **1860** | **1200** | **40** | **11** | **1812.5** | **1000** | **40** | **11** | **1930** | **508** | **70** |
| **15** | **3745** | **1508** | **59.73** | **16** | **1600** | **1003** | **37.31** | **14** | **1845** | **735** | **60** | **15** | **1840** | **1342** | **30** | **12** | **1795** | **950** | **50** | **12** | **1550** | **462** | **70** |
| **16** | **3020** | **1400** | **53.64** | **17** | **1630** | **1004** | **38.4** | **17** | **1215** | **645** | **50** | **18** | **2000** | **1235** | **40** | **13** | **525** | **647.5** | **-20** | **13** | **1475** | **420** | **70** |
| **17** | **2840** | **1224** | **56.9** | **18** | **1640** | **1005** | **38.72** | **18** | **1323** | **860** | **30** | **19** | **1670** | **1420** | **10** | **14** | **575** | **479.5** | **20** | **14** | **2050** | **385** | **80** |
| **18** | **2399** | **1049** | **56.27** | **19** | **1650** | **1006** | **39.03** | **19** | **1170** | **560** | **50** | **20** | **1840** | **1175** | **40** | **15** | **1400** | **350** | **80** | **15** | **890** | **809** | **10** |
| **19** | **1966** | **1204** | **38.76** | **20** | **1920** | **1007** | **47.55** | **20** | **2160** | **980** | **50** | **21** | **1850** | **1045** | **40** | **16** | **1200** | **570.5** | **50** | **16** | **1900** | **700** | **60** |
| **22** | **3200** | **1850** | **42.19** | **23** | **1562** | **1010** | **35.34** | **21** | **1440** | **850** | **40** | **22** | **1820** | **1000** | **50** | **17** | **1450** | **710** | **50** | **17** | **2500** | **644** | **70** |
| **23** | **3400** | **2400** | **29.41** | **24** | **1550** | **1011** | **34.77** | **22** | **960** | **710** | **30** | **23** | **1850** | **1050** | **40** | **18** | **1450** | **700** | **50** | **18** | **900** | **851** | **10** |
| **24** | **3000** | **2000** | **33.33** | **25** | **1660** | **1012** | **39.04** | **25** | **1669.5** | **598.5** | **60** | **26** | **1920** | **1205** | **40** | **19** | **1575** | **750** | **50** | **19** | **2400** | **777** | **70** |
| **25** | **2500** | **1400** | **44.0** | **26** | **1638** | **1013** | **38.16** | **26** | **1417.5** | **902** | **40** | **28** | **1620** | **1430** | **10** | **20** | **1315** | **574** | **60** | **20** | **900** | **662** | **30** |
| **26** | **2680** | **1630** | **39.18** | **27** | **1660** | **1014** | **38.92** | **29** | **1305** | **472.5** | **60** | **29** | **1463** | **1369** | **10** | **21** | **625** | **780** | **-20** | **21** | **3200** | **627** | **80** |
| **27** | **2488** | **1520** | **38.91** | **28** | **1630** | **1015** | **37.73** | **30** | **1017** | **427** | **60** | **30** | **1425** | **1244** | **10** | **22** | **2500** | **497** | **80** | **22** | **2000** | **872** | **60** |
|  | | | | | | | | | | | | **31** | **2600** | **1340** | **50** | **23** | **3000** | **2100** | **30** | **23** | **1380** | **805** | **40** |
|  | | | | | | | | | | | | | | | | **24** | **1200** | **1822** | **-50** | **24** | **2450** | **686** | **70** |
|  | | | | | | | | | | | | | | | | **25** | **2160** | **1654** | **20** | **25** | **1800** | **644** | **60** |
|  | | | | | | | | | | | | | | | | **26** | **1162.5** | **1511** | **-30** | **26** | **1900** | **578** | **70** |
|  | | | | | | | | | | | | | | | | **27** | **2992.5** | **1400** | **50** | **27** | **1000** | **585** | **40** |
|  | | | | | | | | | | | | | | | | **28** | **1620** | **1900** | **-20** | **28** | **2100** | **497** | **80** |
|  | | | | | | | | | | | | | | | | | | | | **29** | **1850** | **490** | **70** |
|  | | | | | | | | | | | | | | | | | | | | **30** | **1900** | **1008** | **50** |
|  | | | | | | | | | | | | | | | | | | | | **31** | **1850** | **956** | **50** |
| **Avg.** | **296832** | **1471409** | **49.35** | **Avg.** | **1674** | **1001.8** | **40.0** | **Avg.** | **1586.5** | **772.7** | **47.7** | **Avg.** | **1847.9** | **1145.5** | **37.7** | **Avg.** | **1460.6** | **889.5** | **34.1** | **Avg.** | **1810.2** | **657.1** | **58.2** |
| **Max.** | **4000** | **2400** | **62.71** | **Max.** | **1920** | **1015** | **47.55** | **Max.** | **2655** | **1365** | **80** | **Max.** | **2600** | **1430** | **50** | **Max.** | **3000** | **2100** | **80** | **Max.** | **3200** | **1008** | **80** |
| **Min.** | **1850** | **1000** | **29.41** | **Min.** | **1550** | **988** | **34.77** | **Min.** | **585** | **280** | **-30** | **Min.** | **1425** | **885** | **10** | **Min.** | **525** | **224** | **-50** | **Min.** | **890** | **385** | **10** |
| **SD** | **597.307** | **298.9934** | **9.847** | **SD** | **90.078** | **8.42578** | **3.285** | **SD** | **537.6447** | **273.5802** | **22.75** | **SD** | **219.7** | **162.9** | **14.122** | **SD** | **693.804** | **565.09** | **37.86848** | **SD** | **560.731** | **170.961** | **20.14** |

**(S 6)**

**Appendix 3: Residual Concentration (RC, mg/l) and Removal Efficiency (RE %) of BOD of AS-Treated Effluents**

| **Oct. 20** | **Oil RC**  **(mg/l)** | | **RE %** | **Nov. 20** | **Oil RC (mg/l)** | | **RE %** | **Dec. 20** | **Oil RC**  **(mg/l)** | | **RE %** | **Jan. 21** | **Oil RC**  **(mg/l)** | | **RE %** | **Feb. 21** | **Oil RC**  **(mg/l)** | | **RE %** | **Mar. 21** | **Oil RC**  **(mg/l)** | | **RE %** |
| --- | --- | --- | --- | --- | --- | --- | --- | --- | --- | --- | --- | --- | --- | --- | --- | --- | --- | --- | --- | --- | --- | --- | --- |
|  | **Infl.** | **Effl.** |  |  | **Infl.** | **Effl.** |  |  | **Infl.** | **Effl.** |  |  | **Infl.** | **Effl.** |  |  | **Infl.** | **Effl.** |  |  | **Infl.** | **Effl.** |  |
| **1** | **1900** | **900** | **52.632** | **1** | **830** | **544** | **34.46** | **1** | **1590** | **770** | **50** | **2** | **1067** | **648** | **40** | **1** | **863** | **630** | **30** | **1** | **938** | **710** | **20** |
| **2** | **2050** | **840** | **59.024** | **2** | **930** | **545** | **41.4** | **2** | **570** | **730** | **-30** | **3** | **1485** | **776** | **50** | **2** | **713** | **263** | **60** | **2** | **1125** | **553** | **50** |
| **3** | **2300** | **1200** | **47.826** | **3** | **860** | **546** | **36.51** | **3** | **850** | **200** | **80** | **4** | **1500** | **556** | **60** | **3** | **488** | **249** | **50** | **3** | **1275** | **478** | **60** |
| **6** | **1500** | **845** | **43.667** | **4** | **800** | **547** | **31.63** | **4** | **840** | **190** | **80** | **5** | **1463** | **760** | **50** | **4** | **956** | **465** | **50** | **4** | **1200** | **387** | **70** |
| **7** | **1965** | **960** | **51.145** | **7** | **739** | **550** | **25.58** | **5** | **1770** | **910** | **50** | **6** | **1376** | **864** | **40** | **5** | **863** | **352** | **60** | **5** | **941** | **412** | **60** |
| **8** | **1900** | **1004** | **47.158** | **8** | **780** | **551** | **29.36** | **6** | **390** | **340** | **10** | **7** | **1373** | **800** | **40** | **6** | **675** | **333** | **50** | **6** | **1575** | **394** | **70** |
| **9** | **1811** | **880** | **51.408** | **9** | **800** | **552** | **31.0** | **9** | **1320** | **560** | **60** | **10** | **1377** | **876** | **40** | **7** | **638** | **202** | **70** | **7** | **1200** | **367** | **70** |
| **10** | **1500** | **741** | **50.6** | **10** | **660** | **553** | **16.21** | **10** | **1460** | **670** | **50** | **11** | **1388** | **708** | **50** | **8** | **581** | **168** | **70** | **8** | **1301** | **273** | **80** |
| **11** | **1000** | **569** | **43.1** | **11** | **740** | **554** | **25.14** | **11** | **1370** | **600** | **60** | **12** | **1373** | **556** | **60** | **9** | **525** | **324** | **40** | **9** | **1485** | **326** | **80** |
| **13** | **1200** | **600** | **50** | **12** | **950** | **555** | **41.58** | **12** | **1320** | **560** | **60** | **13** | **1339** | **720** | **50** | **10** | **1088** | **186** | **80** | **10** | **1425** | **319** | **80** |
| **14** | **1863** | **850** | **54.375** | **15** | **810** | **558** | **31.11** | **13** | **1440** | **650** | **50** | **14** | **1395** | **712** | **50** | **11** | **1359** | **750** | **40** | **11** | **1448** | **330** | **80** |
| **15** | **1750** | **830** | **52.571** | **16** | **710** | **559** | **21.27** | **14** | **1230** | **490** | **60** | **15** | **1380** | **852** | **40** | **12** | **1346** | **713** | **50** | **12** | **1163** | **300** | **70** |
| **16** | **1850** | **762** | **58.811** | **17** | **740** | **560** | **24.32** | **17** | **810** | **430** | **50** | **18** | **1500** | **710** | **50** | **13** | **394** | **486** | **-20** | **13** | **1106** | **273** | **80** |
| **17** | **1500** | **800** | **46.667** | **18** | **750** | **561** | **25.2** | **18** | **880** | **570** | **40** | **19** | **1253** | **650** | **50** | **14** | **431** | **360** | **20** | **14** | **1538** | **250** | **80** |
| **18** | **1620** | **821** | **49.321** | **19** | **760** | **562** | **26.05** | **19** | **780** | **370** | **50** | **20** | **1380** | **642** | **50** | **15** | **1050** | **263** | **70** | **15** | **668** | **526** | **20** |
| **19** | **1340** | **924** | **31.045** | **20** | **1030** | **563** | **45.34** | **20** | **1440** | **650** | **50** | **21** | **1388** | **836** | **40** | **16** | **900** | **428** | **50** | **16** | **1425** | **455** | **70** |
| **22** | **1833** | **1388** | **24.277** | **23** | **672** | **566** | **15.77** | **21** | **960** | **570** | **40** | **22** | **1365** | **800** | **40** | **17** | **1088** | **533** | **50** | **17** | **1875** | **419** | **80** |
| **23** | **1700** | **1000** | **41.176** | **24** | **660** | **567** | **14.09** | **22** | **640** | **470** | **30** | **23** | **1388** | **840** | **40** | **18** | **1088** | **525** | **50** | **18** | **675** | **553** | **20** |
| **24** | **1400** | **963** | **31.214** | **25** | **770** | **568** | **26.23** | **25** | **1110** | **400** | **60** | **26** | **1440** | **964** | **30** | **19** | **1181** | **563** | **50** | **19** | **1800** | **505** | **70** |
| **25** | **1100** | **845** | **23.182** | **26** | **748** | **569** | **23.93** | **26** | **950** | **470** | **50** | **28** | **1215** | **1000** | **20** | **20** | **986** | **431** | **60** | **20** | **675** | **430** | **40** |
| **26** | **1365** | **755** | **44.689** | **27** | **770** | **570** | **25.97** | **29** | **870** | **320** | **60** | **29** | **1097** | **850** | **20** | **21** | **469** | **585** | **-20** | **21** | **2400** | **408** | **80** |
| **27** | **1500** | **796** | **46.933** | **28** | **740** | **571** | **22.84** | **30** | **680** | **280** | **60** | **30** | **1069** | **720** | **30** | **22** | **1875** | **373** | **80** | **22** | **1500** | **567** | **60** |
|  | | | | | | | | | | | | **31** | **1950** | **652** | **70** | **23** | **2250** | **1575** | **30** | **23** | **1035** | **523** | **50** |
|  | | | | | | | | | | | | | | | | **24** | **900** | **1367** | **-50** | **24** | **1838** | **446** | **80** |
|  | | | | | | | | | | | | | | | | **25** | **1620** | **1241** | **20** | **25** | **1350** | **419** | **70** |
|  | | | | | | | | | | | | | | | | **26** | **872** | **1133** | **-30** | **26** | **1425** | **376** | **70** |
|  | | | | | | | | | | | | | | | | **27** | **2244** | **1050** | **50** | **27** | **750** | **380** | **50** |
|  | | | | | | | | | | | | | | | | **28** | **1215** | **1425** | **-20** | **28** | **1575** | **323** | **80** |
|  | | | | | | | | | | | | | | | | | | | | **29** | **1388** | **319** | **80** |
|  | | | | | | | | | | | | | | | | | | | | **30** | **1425** | **655** | **50** |
|  | | | | | | | | | | | | | | | | | | | | **31** | **1388** | **621** | **60** |
| **Avg.** | **1634** | **876** | **45.592** | **Avg.** | **784** | **557.8** | **28.0** | **Avg.** | **1057.7** | **509.1** | **48.6** | **Avg.** | **1386.1** | **765.6** | **44.1** | **Avg.** | **1095.5** | **667.3** | **33.6** | **Avg.** | **1357.8** | **427.1** | **64.5** |
| **Max.** | **2300** | **1388** | **59.024** | **Max.** | **1030** | **571** | **45.34** | **Max.** | **1770** | **910** | **80** | **Max.** | **1950** | **1000** | **70** | **Max.** | **2250** | **1575** | **80** | **Max.** | **2400** | **655** | **80** |
| **Min.** | **1000** | **569** | **23.282** | **Min.** | **660** | **544** | **14.09** | **Min.** | **390** | **190** | **-30** | **Min.** | **1069** | **556** | **20** | **Min.** | **394** | **168** | **-50** | **Min.** | **668** | **250** | **20** |
| **SD** | **317.42** | **172.44** | **9.7** | **SD** | **90.08799** | **8.426** | **8.06** | **SD** | **358.7709** | **180.804** | **22.421** | **SD** | **164.7323** | **114.1397** | **11.93128** | **SD** | **520.33** | **423.767** | **37.36131** | **SD** | **420.551** | **111.064** | **18.52** |

**(S 7)**

**Appendix 4: Residual Concentration (RC, mg/l) and Removal Efficiency (RE %) of TSS of AS-Treated Effluents**

| **Oct. 20** | **Oil RC**  **(mg/l)** | | **RE %** | **Nov. 20** | **Oil RC (mg/l)** | | **RE %** | **Dec. 20** | **Oil RC**  **(mg/l)** | | **RE %** | **Jan. 21** | **Oil RC**  **(mg/l)** | | **RE %** | **Feb. 21** | **Oil RC**  **(mg/l)** | | **RE %** | **Mar. 21** | **Oil RC**  **(mg/l)** | | **RE %** |
| --- | --- | --- | --- | --- | --- | --- | --- | --- | --- | --- | --- | --- | --- | --- | --- | --- | --- | --- | --- | --- | --- | --- | --- |
|  | **Infl.** | **Effl.** |  |  | **Infl.** | **Effl.** |  |  | **Infl.** | **Effl.** |  |  | **Infl.** | **Effl.** |  |  | **Infl.** | **Effl.** |  |  | **Infl.** | **Effl.** |  |
| **1** | **250** | **74** | **70.4** | **1** | **300** | **137** | **54.333** | **1** | **390** | **150** | **60** | **2** | **179** | **67** | **60** | **1** | **210** | **110** | **50** | **1** | **238** | **196** | **20** |
| **2** | **200** | **61** | **69.5** | **2** | **400** | **237** | **40.75** | **2** | **230** | **110** | **50** | **3** | **310** | **45** | **90** | **2** | **200** | **94** | **50** | **2** | **285** | **131** | **50** |
| **3** | **192** | **71** | **63.021** | **3** | **330** | **167** | **49.394** | **3** | **269** | **100** | **60** | **4** | **330** | **119** | **60** | **3** | **171** | **74** | **60** | **3** | **323** | **100** | **70** |
| **6** | **180** | **74** | **58.889** | **4** | **270** | **107** | **60.37** | **4** | **267** | **130** | **50** | **5** | **280** | **145** | **50** | **4** | **145** | **79** | **50** | **4** | **304** | **162** | **50** |
| **7** | **235** | **79** | **66.383** | **7** | **209** | **46** | **77.99** | **5** | **290** | **190** | **30** | **6** | **165** | **52** | **70** | **5** | **132** | **62** | **50** | **5** | **238** | **120** | **50** |
| **8** | **244** | **86** | **64.754** | **8** | **250** | **87** | **65.2** | **6** | **344** | **180** | **50** | **7** | **160** | **50** | **70** | **6** | **145** | **59** | **60** | **6** | **399** | **125** | **70** |
| **9** | **201** | **82** | **59.204** | **9** | **270** | **107** | **60.37** | **9** | **120** | **60** | **50** | **10** | **166** | **64** | **60** | **7** | **218** | **111** | **50** | **7** | **304** | **122** | **60** |
| **10** | **164** | **74** | **54.878** | **10** | **130** | **33** | **74.615** | **10** | **260** | **50** | **80** | **11** | **180** | **82** | **50** | **8** | **152** | **74** | **50** | **8** | **330** | **114** | **70** |
| **11** | **179** | **66** | **63.128** | **11** | **210** | **47** | **77.619** | **11** | **170** | **58** | **70** | **12** | **160** | **94** | **40** | **9** | **158** | **88** | **40** | **9** | **376** | **136** | **60** |
| **13** | **155** | **68** | **56.129** | **12** | **420** | **257** | **38.81** | **12** | **120** | **60** | **50** | **13** | **115** | **42** | **60** | **10** | **234** | **96** | **60** | **10** | **361** | **133** | **60** |
| **14** | **137** | **59** | **56.934** | **15** | **280** | **117** | **58.214** | **13** | **240** | **30** | **90** | **14** | **190** | **70** | **60** | **11** | **430** | **90** | **80** | **11** | **367** | **138** | **60** |
| **15** | **193** | **52** | **73.057** | **16** | **180** | **17** | **90.556** | **14** | **240** | **81** | **70** | **15** | **170** | **56** | **70** | **12** | **393** | **175** | **60** | **12** | **295** | **125** | **60** |
| **16** | **213** | **67** | **68.545** | **17** | **210** | **47** | **77.619** | **17** | **360** | **90** | **80** | **18** | **330** | **65** | **80** | **13** | **166** | **142** | **10** | **13** | **280** | **114** | **60** |
| **17** | **200** | **65** | **67.5** | **18** | **220** | **57** | **74.091** | **18** | **320** | **50** | **80** | **19** | **150** | **51** | **70** | **14** | **211** | **100** | **50** | **14** | **390** | **105** | **70** |
| **18** | **180** | **71** | **60.556** | **19** | **230** | **67** | **70.87** | **19** | **250** | **74** | **70** | **20** | **170** | **62** | **60** | **15** | **162** | **102** | **40** | **15** | **169** | **219** | **-30** |
| **19** | **184** | **80** | **56.522** | **20** | **500** | **337** | **32.6** | **20** | **240** | **30** | **90** | **21** | **180** | **45** | **80** | **16** | **108** | **84** | **20** | **16** | **361** | **190** | **50** |
| **22** | **217** | **74** | **65.899** | **23** | **142** | **34** | **76.056** | **21** | **210** | **63** | **70** | **22** | **150** | **74** | **50** | **17** | **213** | **88** | **60** | **17** | **475** | **175** | **60** |
| **23** | **182** | **78** | **57.143** | **24** | **130** | **60** | **53.846** | **22** | **200** | **100** | **50** | **23** | **180** | **65** | **60** | **18** | **205** | **82** | **60** | **18** | **171** | **231** | **-40** |
| **24** | **234** | **79** | **66.239** | **25** | **240** | **77** | **67.917** | **25** | **180** | **140** | **20** | **26** | **250** | **74** | **70** | **19** | **243** | **79** | **70** | **19** | **456** | **211** | **50** |
| **25** | **249** | **75** | **69.88** | **26** | **218** | **55** | **74.771** | **26** | **250** | **95** | **60** | **28** | **130** | **95** | **30** | **20** | **111** | **64** | **40** | **20** | **266** | **180** | **30** |
| **26** | **241** | **74** | **69.295** | **27** | **240** | **77** | **67.917** | **29** | **362** | **100** | **70** | **29** | **220** | **115** | **50** | **21** | **265** | **50** | **80** | **21** | **608** | **170** | **70** |
| **27** | **255** | **88** | **65.49** | **28** | **210** | **47** | **77.619** | **30** | **351** | **163** | **50** | **30** | **200** | **115** | **40** | **22** | **153** | **74** | **50** | **22** | **380** | **117** | **70** |
|  | | | | | | | | | | | | **31** | **330** | **197** | **40** | **23** | **225** | **88** | **60** | **23** | **262** | **119** | **50** |
|  | | | | | | | | | | | | | | | | **24** | **204** | **80** | **60** | **24** | **466** | **126** | **70** |
|  | | | | | | | | | | | | | | | | **25** | **292** | **57** | **80** | **25** | **342** | **124** | **60** |
|  | | | | | | | | | | | | | | | | **26** | **213** | **155** | **30** | **26** | **361** | **120** | **70** |
|  | | | | | | | | | | | | | | | | **27** | **230** | **157** | **30** | **27** | **190** | **123** | **40** |
|  | | | | | | | | | | | | | | | | **28** | **210** | **162** | **20** | **28** | **399** | **125** | **70** |
|  | | | | | | | | | | | | | | | | | | | | **29** | **352** | **134** | **60** |
|  | | | | | | | | | | | | | | | | | | | | **30** | **361** | **124** | **70** |
|  | | | | | | | | | | | | | | | | | | | | **31** | **352** | **128** | **60** |
| **Avg.** | **203.864** | **72.5909** | **63.788** | **Avg.** | **254** | **100.8** | **64.6** | **Avg.** | **257.4** | **95.6** | **61.4** | **Avg.** | **205.3** | **80.8** | **59.5** | **Avg.** | **218** | **99.9** | **50** | **Avg.** | **348.4** | **146.9** | **50.9** |
| **Max.** | **255** | **88** | **73.057** | **Max.** | **500** | **337** | **90.556** | **Max.** | **390** | **190** | **90** | **Max.** | **330** | **197** | **90** | **Max.** | **430** | **175** | **80** | **Max.** | **608** | **231** | **70** |
| **Min.** | **137** | **52** | **54.878** | **Min.** | **130** | **17** | **32.6** | **Min.** | **120** | **30** | **20** | **Min.** | **115** | **42** | **30** | **Min.** | **108** | **50** | **10** | **Min.** | **169** | **105** | **-40** |
| **SD** | **32.5287** | **8.49951** | **5.3325** | **SD** | **90.07799** | **79.97** | **14.515** | **SD** | **73.6155** | **45.44982** | **17.39787** | **SD** | **66.50166** | **37.23016** | **14.60924** | **SD** | **75.80657** | **34.77056** | **19.30615** | **SD** | **101.286** | **36.7439** | **29.06** |

(Supplementary Table)

S 8: Residual Concentration (RC, mg/l) and Removal Efficiency (RE%) of the Tested Contaminants

in the Domestic Wastewater After Treatment Using Unmodified AS Reactor

| Time (Days) | Temp. (ºC) | pH | DO | TSS | | TDS | | BOD | | COD | | OG | | |
| --- | --- | --- | --- | --- | --- | --- | --- | --- | --- | --- | --- | --- | --- | --- |
|  |  |  | (mg/l) | RC | RE | **RC** | **RE** | **RC** | **RE** | **RC** | **RE** | **RC** | **RE** |  |
| **Raw Influent** | **20.5±0.42** | **7.2±0.57** | **3.5±0.72** | **280±0.09** |  | **3000±0.34** |  | **1600±0.66** |  | **2900±0.37** |  | **215±0.43** |  |  |
| 1 | **20.8±0.57** | **7.2±0.61** | **4.61±0.72** | **175±0.37** | **37.5±0.37*** | **2974±0.895** | **0.87±0.895*** | **1425±0.34** | **10.94±0.34*** | **2400±0.69** | **17.24±0.69*** | **204±0.28** | **5.12±0.28*** |  |
| 2 | **21.5±0.89** | **7.1±0.87** | **5.56±0.64** | **161±0.12** | **42.5±0.12** | **2951±0.04** | **1.63±0.04** | **1300±1.38** | **18.75±1.38** | **2100±0.79** | **27.58±0.79** | **200±0.15** | **7.5±0.15** |  |
| 4 | **19.7±0.37** | **7.2±0.43** | **5.66±0.92** | **143±0.77** | **48.9±0.77** | **2932±0.03** | **2.27±0.03** | **1100±1.12** | **31.25±1.12** | **1890±0.09** | **34.83±0.09** | **195±0.01** | **9.3±0.01** |  |
| 5 | **20.8±0.61** | **7.1±0.57** | **5.42±0.89** | **142±0.63** | **49.3±0.63** | **2899±0.75** | **3.37±0.75** | **900±0.75** | **43.75±0.75** | **1720±0.86** | **40.69±0.86** | **181±0.61** | **15.81±0.61** |  |
| 6 | **21.3±0.34** | **7.1±0.66** | **4.37±0.37** | **132±0.43** | **52.9±0.43** | **2887±0.34** | **3.77±0.34** | **842±0.66** | **47.38±0.66** | **1600±0.37** | **44.83±0.37** | **167±0.43** | **22.33±0.43** |  |
| 7 | **21.5±0.28** | **7.3±0.13** | **4.45±0.26** | **125±0.32** | **55.4±0.32** | **2871±0.38** | **4.3±0.38** | **712±0.34** | **55.5±0.34** | **1400±0.38** | **51.72±0.38** | **154±0.57** | **32.56±0.57** |  |
| 9 | **19.9±0.57** | **7.6±0.63** | **4.62±0.66** | **122±0.54** | **56.4±0.54** | **2853±0.52** | **4.9±0.52** | **650±0.98** | **59.38±0.98** | **1200±0.63** | **58.62±0.63** | **110±0.84** | **48.83±0.84** |  |
| 11 | **19.9±0.37** | **7.2±0.43** | **5.81±0.92** | **114±0.77** | **59.3±0.77** | **2824±0.03** | **5.87±0.03** | **610±0.12** | **61.88±0.12** | **1140±0.09** | **60.69±0.09** | **104±0.01** | **51.63±0.01** |  |
| 12 | **20.1±0.61** | **7.1±0.93** | **4.73±0.71** | **109±0.95** | **61.1±0.95**** | **2812±0.41** | **6.27±0.41**** | **575±0.99** | **64.10±0.99**** | **1100±0.86** | **62.1±0.86**** | **92±0.29** | **57.21±0.29**** |  |

*** The Lowest and ** The highest RE%**

(Supplementary Table)

S 9: Residual Concentration (RC, mg/L) and Removal Efficiency (RE%) of the Tested Contaminants

in the Industrial Wastewater After Treatment Using Unmodified AS Reactor

| Time (Days) | Temp. (ºC) | pH | DO | TSS | | TDS | | BOD | | COD | | OG | |
| --- | --- | --- | --- | --- | --- | --- | --- | --- | --- | --- | --- | --- | --- |
|  |  |  | (mg/l) | RC | RE | **RC** | **RE** | **RC** | **RE** | **RC** | **RE** | **RC** | **RE** |
| Raw Influent | 20.8±0.63 | 7.21±0.32 | **4.8±0.26** | 300±0.38 |  | 3100±0.34 |  | 1200±0.38 |  | 2342±0.57 |  | 380±0.61 |  |
| 1 | 20.7±0.43 | 7.11±0.01 | **4.8±0.32** | 250±0.65 | 16.7±0.65* | 3075±0.75 | 0.81±0.75* | 1120±0.36 | 6.67±0.36* | 2210±0.89 | 5.64±0.89* | 340±0.87 | 10.53±0.87* |
| 2 | 20.9±0.32 | 7.13±0.72 | **4.91±0.32** | 231±0.09 | 23±0.09 | 2710±0.34 | 12.6±0.34** | 1015±0.66 | 15.42±0.66 | 2040±0.37 | 12.89±0.37 | 321±0.43 | 15.53±0.43 |
| 3 | 21.7±0.75 | 7.12±0.01 | **4.52±0.75** | 211±0.81 | 29.7±0.81 | 2810±0.46 | 9.35±0.46 | 920±0.49 | 23.33±0.49 | 1760±0.61 | 24.85±0.61 | 300±0.57 | 21.05±0.57 |
| 4 | 21.7±0.66 | 7.11±0.99 | **4.4±0.61** | 198±0.35 | 34±0.35 | 2834±0.35 | 8.58±0.35 | 870±0.07 | 27.5±0.07 | 1650±3.17 | 29.55±3.17 | 240±0.36 | 36.84±0.36 |
| 6 | 20.0±0.61 | 7.13±0.71 | **4.41±0.87** | 182±0.95 | 39.3±0.95 | 2847±0.41 | 8.16±0.41 | 820±0.99 | 31.67±0.99 | 1530±0.86 | 34.67±0.86 | 220±0.29 | 42.11±0.29 |
| 7 | 20.0±0.12 | 7.21±0.69 | **4.41±0.43** | 170±0.64 | 43.3±0.64 | 2893±0.73 | 6.68±0.73 | 740±0.46 | 38.33±0.46 | 1560±0.57 | 33.39±0.57 | 189±0.63 | 50.26±0.63 |
| 8 | 20.3±0.42 | 7.22±0.72 | **5.05±0.57** | 160±0.09 | 46.7±0.09 | 2811±0.34 | 9.32±0.34 | 700±0.66 | 41.67±0.66 | 1411±0.37 | 39.75±0.37 | 176±0.43 | 53.68±0.43 |
| 13 | 20.4±0.57 | 7.24±0.99 | **5.6±0.66** | 134±0.35 | 55.3±0.35** | 2877±0.35 | 7.19±0.35 | 600±0.07 | 50±0.07** | 1400±0.17 | 40.22±0.17** | 164±0.36 | 56.84±0.36** |

*** The Lowest and ** The highest RE%**

(Supplementary Table)

S 10: Residual Concentration (RC, mg/L) and Removal Efficiency (RE%) of the Tested Contaminants

in the Industrial Oily Wastewater After Treatment Using AS Reactor Modified with Fe_3_O_4_/Si NPs Composite

| Time (Days) | Temp. (ºC) | pH | DO  (mg/l) | TSS | | TDS | | BOD | | COD | | OG | |
| --- | --- | --- | --- | --- | --- | --- | --- | --- | --- | --- | --- | --- | --- |
|  |  |  |  | RC | RE | **RC** | **RE** | **RC** | **RE** | **RC** | **RE** | **RC** | **RE** |
| Raw Influent | 25.1±0.61 | 6.98±0.42 | 4.1 ±0.44 | 300±0.57 |  | 2500±0.89 |  | 1700±0.37 |  | 2900±0.41 |  | 480±0.34 |  |
| 1 | 24.0±0.93 | 7.1±0.57 | 4.26±0.34 | 250±0.66 | 16.67±0.66 | 2410±0.87 | 3.6±0.87 | 1340±0.43 | 21.18±0.43 | 2400±0.57 | 17.24±0.57 | 361±0.03 | 24.79±0.03 |
| 2 | 26.1±0.71 | 7.1±0.72 | 3.75±0.38 | 200±0.99 | 33.33±0.99 | 2465±0.44 | 1.4±0.44 | 1120±0.92 | 34.12±0.92 | 2150±0.89 | 25.86±0.89 | 285±0.12 | 40.63±0.12 |
| 4 | 24.5±0.95 | 7.11±0.09 | **3.83±0.12** | 185±0.35 | 38.33±0.35 | 2477±0.12 | 0.92±0.12 | 844±0.77 | 50.35±0.77 | 1743±0.43 | 39.9±0.43 | 204±0.43 | 57.5±0.43 |
| 5 | 25.6±0.41 | 7.14±0.34 | **3.45±0.75** | 162±0.35 | 46.0±0.35 | 2420±0.28 | 3.2±0.28 | 624±0.49 | 63.29±0.49 | 1380±0.72 | 52.41±0.72 | 142±0.52 | 70.42±0.52 |
| 6 | 25.1±0.99 | 7.13±0.66 | 4.31±0.44 | 124±0.07 | 58.67±0.07 | 2463±0.41 | 1.48±0.41 | 420±0.44 | 75.29±0.44 | 1150±0.09 | 60.34±0.09 | 103±0.98 | 78.54±0.98 |
| 7 | 25.6±0.86 | 7.12±0.37 | 4.47±0.34 | 101±0.17 | 66.33±0.17 | 2425±0.99 | 3.0±0.99 | 300±0.73 | 82.35±0.73 | 950±0.34 | 67.24±0.34 | 67±0.43 | 86.04±0.43 |
| 8 | 25.5±0.29 | 7.0±0.43 | **4.96±0.98** | 65±0.36 | 78.33±0.36 | 2466±0.32 | 1.36±0.32 | 210±0.44 | 87.65±0.44 | 430±0.44 | 85.17±0.44 | 34±0.84 | 92.92±0.84 |

*** The Lowest and ** The highest RE%**
